# Multi-modal image analysis for semi-automatic segmentation of the total liver and liver arterial perfusion territories for radioembolization

**DOI:** 10.1186/s13550-019-0485-x

**Published:** 2019-02-20

**Authors:** Esmaeel Jafargholi Rangraz, Walter Coudyzer, Geert Maleux, Kristof Baete, Christophe M. Deroose, Johan Nuyts

**Affiliations:** 1 Nuclear Medicine, Department of imaging and pathology, UZ & KU Leuven, Leuven, Belgium; 2 Radiology Section, Department of imaging and pathology, UZ Leuven, Leuven, Belgium; 3 Radiology Section, Department of imaging and pathology, UZ & KU Leuven, Leuven, Belgium

**Keywords:** Radioembolization, Selective internal radiation therapy (SIRT), Transarterial radioembolization (TARE), Liver segmentation, Liver lobe segmentation, Liver perfusion territory segmentation, CBCT

## Abstract

**Purpose:**

We have developed a multi-modal imaging approach for SIRT, combining ^99m^Tc-MAA SPECT/CT and/or ^90^Y PET, ^18^F-FDG PET/CT, and contrast-enhanced CBCT for voxel-based dosimetry, as a tool for treatment planning and verification. For radiation dose prediction calculations, a segmentation of the total liver volume and of the liver perfusion territories is required.

**Method:**

In this paper, we proposed a procedure for multi-modal image analysis to assist SIRT treatment planning. The pre-treatment ^18^F-FDG PET/CT, ^99m^Tc-MAA SPECT/CT, and contrast-enhanced CBCT images were registered to a common space using an initial rigid, followed by a deformable registration. The registration was scored by an expert using Likert scores. The total liver was segmented semi-automatically based on the PET/CT and SPECT/CT images, and the liver perfusion territories were determined based on the CBCT images. The segmentations of the liver and liver lobes were compared to the manual segmentations by an expert on a CT image.

**Result:**

Our methodology showed that multi-modal image analysis can be used for determination of the liver and perfusion territories using CBCT in SIRT using all pre-treatment studies. The results for image registration showed acceptable alignment with limited impact on dosimetry.

The image registration performs well according to the expert reviewer (scored as perfect or with little misalignment in 94% of the cases). The semi-automatic liver segmentation agreed well with manual liver segmentation (dice coefficient of 0.92 and an average Hausdorff distance of 3.04 mm). The results showed disagreement between lobe segmentation using CBCT images compared to lobe segmentation based on CT images (average Hausdorff distance of 14.18 mm), with a high impact on the dosimetry (differences up to 9 Gy for right and 21 Gy for the left liver lobe).

**Conclusion:**

This methodology can be used for pre-treatment dosimetry and for SIRT planning including the determination of the activity that should be administered to achieve the therapeutical goal. The inclusion of perfusion CBCT enables perfusion-based definition of the liver lobes, which was shown to be markedly different from the anatomical definition in some of the patients.

## Introduction

Radioembolization (RE), also known as selective internal radiation therapy (SIRT) or transarterial RE (TARE), is a promising therapy in both safety and efficacy aspects for non-resectable primary and metastatic liver malignancies [1–5] which is recommended in guidelines in the salvage setting in metastatic colorectal cancer (mCRC) and hepatocellular carcinoma (HCC) when other therapies are contraindicated or have failed, and for small tumors in patients waiting for liver transplantation [6, 7]. Several large randomized trials have investigated SIRT in HCC and mCRC and did not meet their primary endpoint, highlighting the need for optimization of the technique and patient selection [1, 2, 8–11].

In SIRT, millions of implantable microspheres containing yttrium-90 (^90^Y) are administrated into the hepatic artery during a femoral arterial catheterization [12, 13] which results in higher concentration in the tumors located within that liver perfusion territory than within the normal liver parenchyma [14].

Before injecting the ^90^Y microspheres, a simulation is performed in which the patient-specific vascular anatomy is determined and specific arteries are coiled to prevent extra-hepatic dissemination [15]. Then, macro-aggregated albumin (MAA) particles labeled with technetium-99m (^99m^Tc) are injected, and within the hour after injection, a scintigraphic planar imaging and single-photon emission-computed tomography with X-ray computed tomography (SPECT/CT) are performed [16]. These images are used to quantify a possible pulmonary shunt and determine extra-hepatic uptake. These SPECT/CT images can be used to predict the intrahepatic distribution of the ^90^Y spheres as well, enabling a pre-therapeutic dosimetric analysis. Subsequently, the patient undergoes a second procedure in which a prescribed amount of ^90^Y spheres are injected. ^90^Y is a beta emitter. In soft tissue, 2.23% of the electrons produce a bremsstrahlung photon with an energy of 50 keV or more. Its decay also has a very small positron branching ratio (32 per million decays) [17, 18]. Therefore, the actual distribution of the spheres can be determined by bremsstrahlung emission-computed tomography or time-of-flight positron emission tomography (PET) [19–21].

Because of the high kinetic energy of the emitted electrons (mean energy of 0.934 MeV, mean and maximum tissue penetration of 2.5, and 11 mm, respectively), treatment with ^90^Y resin microspheres can achieve an absorbed dose (the energy that will be absorbed per unit of tissue mass) of about 100 to 1000 Gy to the tumor which is sufficient for complete tumor ablation, while keeping the healthy tissue irradiation below the safety threshold (typically in the order of 30–50 Gy) [22, 23]. Estimating the absorbed dose to the tumor(s) and the normal liver parenchyma has a key role in radionuclide therapy for predicting the tumor response and healthy liver toxicity [24].

The so-called partition method is a tissue level dosimetry method that is used widely as a tool to predict mean absorbed dose in the tumor and healthy liver. This method assumes uniform activity in tumor and normal liver compartments [25]. However, there are some concerns about using the mean absorbed dose in SIRT because of the inherent activity heterogeneity. For this reason, using a more detailed dosimetry is vital for treatment optimization [8, 26]. The role of MAA-based dose estimation for a safe normal parenchyma absorbed dose and efficient tumor absorbed dose has been investigated in several studies [27–29]. Post-treatment dosimetry has also been studied to verify the treatment planning [30–33].

Recent arguments over choosing between an activity prescription based on body surface area (BSA) and dosimetry-based prescription in the treatment planning procedure [34, 35] accentuate the relevance of quality control, dosimetry process verification, and standardization in the field of SIRT. In addition to the activity distribution estimation uncertainties (e.g., simulating power of MAA, partial volume effect, breathing motion), some errors are introduced by image processing techniques, e.g., image registration and segmentation, which need to be avoided [8]. To date, activity calculation based on BSA is used for SIRT with resin microspheres in one third of European hospitals regardless of availability of numerous commercial softwares designed for dosimetry [36, 37]. This is possibly due to lack of image processing validation, error estimation, or complexity of the process and regulation.

Image registration accuracy has been investigated for CT to CT liver registration for contrast-enhanced diagnostic CTs [38]. Over the past decade, numerous semi-automatic and automatic approaches for liver segmentation [39, 40] on CT that rely on histogram-based methods [41, 42], graph cut [43–45], region growing [45–47], geometric deformable model and level set [48–50], probabilistic atlas [51, 52], statistical shape models [53–55], and recently neural network [56–59] have been proposed. Despite these efforts, image registration and segmentation remains a challenging task for SIRT application for several reasons: (1) liver is a soft tissue and liver shape is heavily dependent on patient positioning (e.g., the position of the arms); (2) the liver shape in SIRT patients differs from the normal shape, because of preceding treatments (liver resection, liver ablation, chemotherapy) and tumor growth which makes it challenging to use liver segmentation techniques which are dependent on the liver shape for these patients; (3) liver is a soft tissue and its Hounsfield units are similar to those of adjacent organs like the heart, spleen, stomach, and kidney, which makes liver segmentation on non-contrast-enhanced CTs (e.g., CT from MAA study) hard, even for experts; (4) CT from MAA study is not a dedicated diagnostic CT, this low-dose CT usually suffers from streak artifacts; and (5) the interval between the MAA study and the diagnostic high-dose, contrast-enhanced CT from from fluorine-18 fluorodeoxyglucose (^18^F-FDG) PET/CT study can be up to weeks to even 1 or 2 months and the liver can deform dramatically over time for several reasons, e.g., tumor change.

Although liver segmentation techniques offer highly accurate results for healthy liver on contrast-enhanced CTs, few studies applied volumetric methods to the baseline and/or non-contrast-enhanced, low-dose CTs for radiation therapy planning. Monsky et al. propose a method based on iterative watershed segmentation to semi-automatic volumetric segmentation of the liver, tumor, and necrosis using multiphase CTs in 14 patients. They compare their results with manual liver segmentation and report a good interobserver/intraobserver reproducibility [60]. Goryawala et al. present a framework for extracting a 3D liver segmentation based on coupling a *k*-mean algorithm with a localized contouring method. They applied their method to 5 patients aiming at minimizing human intervention [61]. In [62], and they improved their method by decreasing the human intervention time and applying their workflow to 34 patients with liver metastatic.

To overcome the inaccuracy of liver segmentation due to the similar Hounsfield values of the liver and its surrounding organs in non-contrast-enhanced CTs, some studies use co-segmentation algorithm, using information from different co-registered modalities (e.g., PET and CT) to guide the liver segmentation. Wang et al. use ^18^F-FDG uptake difference between the liver and adjacent organs to separate them for 12 patients [63]. Later, they apply their method to more patients [64] and using probabilistic atlas [65]. Mendes et al. present a framework for outlining liver using CT alone, PET alone, and a hybrid modality liver segmentation using information from PET and CT together [66].

The main aim of this study is to develop a multi-modal image analysis approach that can be used for voxel level and partition model dosimetry, using pre-treatment simulation based on the ^99m^Tc-MAA study. This methodology is generic and can be extended for post-treatment dose verification images. For this purpose, we developed and evaluated a new methodology for image registration, for liver segmentation using all pre-therapy image data, for ^18^F-FDG tumor segmentation using ^18^F-FDG PET/CT, and for liver perfusion territory (LPT) segmentation using the cone beam CT (CBCT).

To our best knowledge, this is the first attempt to investigate the role of lobe segmentation based on contrast-enhanced CBCT for dosimetry and activity prescription. LPTs are usually segmented using the anatomical landmarks on CT, which reflect the standard anatomical venous lobe segmentation [67]. In our hospital, contrast-enhanced CBCT is acquired in the early and late arterial phase during the angiographic work-up to outline the different LPTs [68, 69].

To our knowledge, there are no reported studies investigating the registration accuracy for SIRT therapy planning; CBCT and low-dose, non-contrast-enhanced CT registration. We are also implementing a multi-model liver segmentation approach which uses the ^18^F-FDG-PET/CT aligned to the CT from the MAA-SPECT/CT study. The current version is still semi-automatic, and its preliminary evaluation shows that it produces a segmentation similar to that produced by an expert operator on the contrast-enhanced CT using commercial software. Nevertheless, we present the method already here because we believe that such a multi-modal segmentation approach has the potential to improve the segmentation of the liver and the tumor lesions. Details about the method are provided as supplementary material.

The paper is organized as follows: In the “Methods and materialsMethods” section, the multi-modal image analysis algorithm is described: first, all images are registered to the same space, then the liver, the LPTs and the tumors are delineated. Then, the results of the proposed algorithm are shown and compared to a manual segmentation by an expert in the “Results” section. Finally, in “Discussion” section, our results are discussed. In the “Conclusion” section, some conclusions are presented and some future directions are discussed.

## Methods and materials

### Patient selection

Between May 2014 and December 2017, 22 consecutive patients underwent bilobar SIRT in the University Hospitals Leuven (UZ Leuven, Leuven, Belgium) with early and late arterial phase CBCT imaging for both lobes before treatment (for delineating different LPTs) and with ^18^F-FDG PET/CT imaging (for delineating ^18^F-FDG avid malignancies). Of these, 5 patients were excluded from the study due to artifacts or low-image quality in the MAA SPECT/CT study (*n* = 3), CBCT images (*n* = 1) and ^18^F-FDG PET/CT study (*n* = 1). Pre- and post- treatment images were collected and analyzed retrospectively. From these patients, a set of 7 patients with a HCC, 2 breast, 2 melanoma, a colorectal, and an esophageal cancer was arbitrarily selected to optimize the parameters of the algorithm (so-called “training set”) and 10 patients, 6 colorectal cancer, an esophageal cancer, a neuroendocrine tumor, a colon cancer, and a cholangiocellular cancer were used for an independent evaluation (so-called “test set”). Detailed patient characteristics are provided in the Appendix.

### Pre-treatment studies

**Fig. 1:**
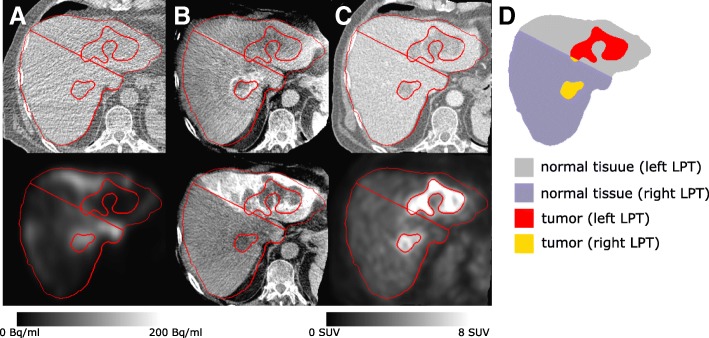
Single transaxial slice of radioembolization pre-treatment studies and segmentation result (patient ID BI01 from training set in our database): **a** the CT and activity image of the ^99m^Tc-MAA SPECT/CT scan. Correction for scatter and attenuation is applied and a phantom-based calibration factor is used to obtain images of absolute ^99m^Tc concentration [kBq/cc]. **b** CBCT images with contrast enhancement facilitating delineation of the right and left liver lobe. **c** The CT and PET of the ^18^F-FDG PET/CT study. **d** The segmented liver, tumor, and LPTs

Pre-treatment studies were performed for SIRT (see Fig. 1) based on the European Association of Nuclear Medicine (EANM) guideline [70], the recommendations of the American Association of Physics in Medicine (AAPM) [13], and the SIRTEX manual [71].

For a SIRT simulation, about 150 MBq of ^99m^Tc-MAA was infused into the hepatic artery at the position where the therapeutic activity was expected to be administered (about 100 and 50 MBq to the vessels that feed right and left LPTs, respectively). A SPECT/CT was performed as soon as possible [16], typically within 1 h on a Symbia T16 camera (Siemens Healthineers, Erlangen, Germany).

SPECT, using a dual-head gamma camera, was performed with rotation over 180 °, 60 views per detector, and 21 s per view in a 128 ×128 matrix with 15% energy window centered at the photopeak of ^99m^Tc (140 kev) using low-energy high-resolution collimators. The reconstruction was done using the ordered subset expectation maximization (OSEM) algorithm accounting for attenuation, position-dependent collimator blurring, and a scatter contribution, which was estimated using a dual-energy scatter window. Phantom experiments were done to determine the calibration factor (producing a system sensitivity of 11.7 cpm/kBq), which absolutely quantifies the reconstruction of the images, these results are in line with results provided by Zeintl et al. [72]. This image was reconstructed with an isotropic voxel size of 4.8 mm. The CT scan (120 mAs, 110 kV) was acquired with 0.9 mm in plane voxel size and 1.0-mm slice thickness.

During the angiographic work-up, contrast-enhanced CBCTs were acquired in the early and late arterial phase, outlining the LPTs of the hepatic artery branches. CBCTs were performed using XtraVision (Philips Healthcare, Amsterdam, Netherlands). These images are not mentioned in the guidelines and there are not many centers that acquire these images as part of the SIRT procedure.

For some patients (e.g., patients with ^18^F-FDG-avid tumors), a whole body ^18^F-FDG PET/CT with a Biograph 40 TruePoint TrueV system (Siemens Healthineers, Erlangen, Germany) was performed up to 2 months before treatment to provide the tumor metabolic data. CT imaging was done mostly with intravenous contrast enhancement for outlining the total liver and the liver tumor burden (85 mAs, 120 kV, plane voxel size of 0.98 mm, slice thickness of 1.5 mm). The PET images were reconstructed using resolution recovery, attenuating and scatter correction, and voxel size, and slice thickness are 2.9 and 5 mm respectively.

### SIRT procedure

SIR-Spheres (SIRTEX Medical Ltd., Sidney, Australia) were used in all patients. According to the resin microspheres’ manufacturer guideline (SIRTEX Medical Ltd., Australia, NSW), determination of therapeutic activity was based on the BSA method [71] or a one-compartment partition model aiming at keeping the dose to each lobe below 40 to 50 Gy [73]. The activity was adjusted to have a lung absorbed dose below 30 Gy. The calculated activity was administered according to the manufacturer recommendation [71].

### Image processing

We proposed a multi-modal image analysis approach for registering all pre-treatment images to an identical space. These aligned images were used for semi-automatic total liver, tumor, and LPT delineation. All codes were written in-house using IDL 8.4 (Harris Geospatial Solution, Boulder, CO, USA).

**Fig. 2:**
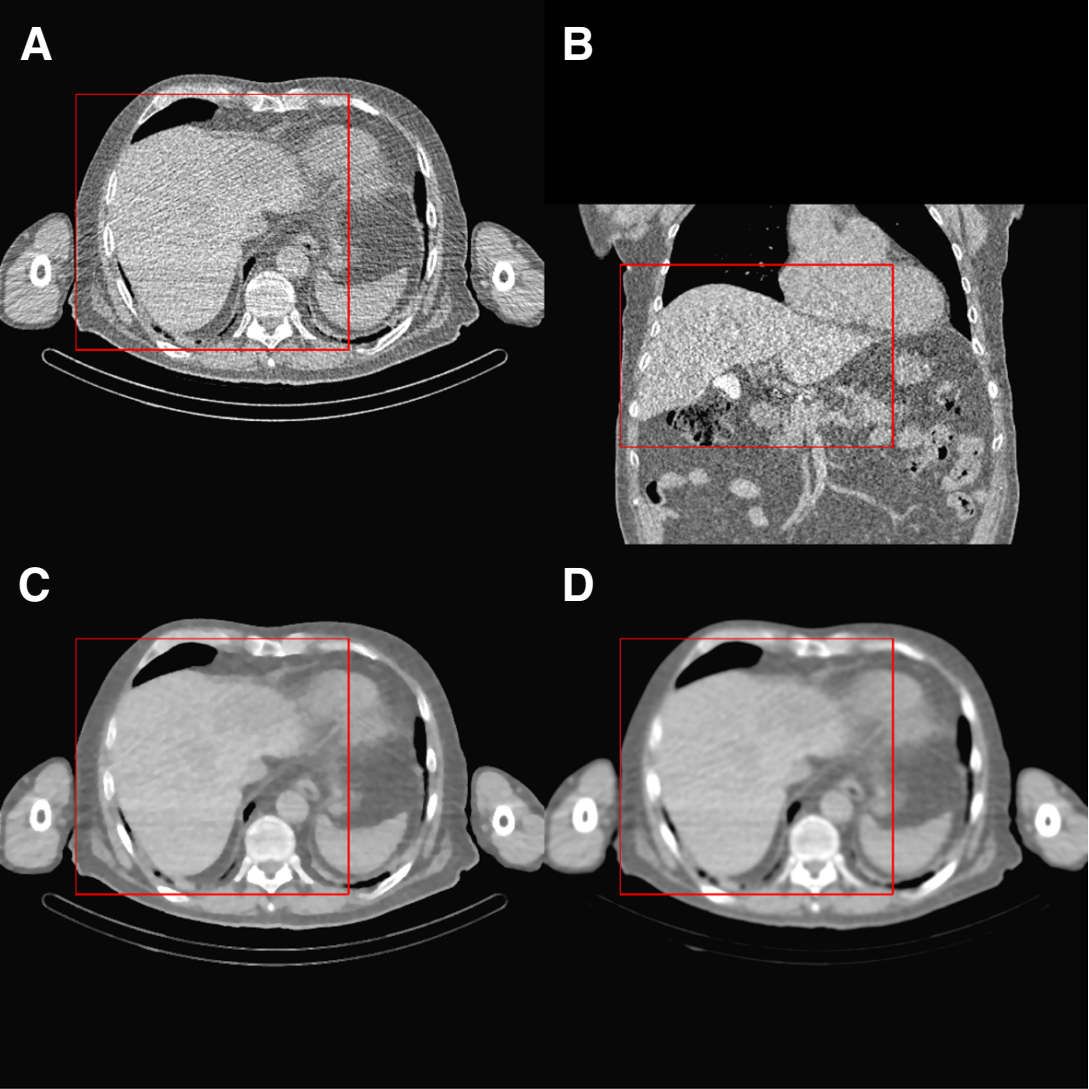
Patient ID BI01 from training set in our database: **a**, **b** transaxial and coronal view of a CT_MAA_, **c**, **d** median filter and Gaussian filter (fwhm = 4) of the same slide in transaxial slice (image **c**is sharper and voxels within the liver are more homogeneous; red contours show the boxes that are defined for data cropping)

Before registering all images to an identical space, a box was defined manually for each image which contains the entire liver; this makes the whole procedure faster and also gives a data reduction for the image registration and segmentation. To define the box for each image, the first and the last plane that contain the liver were selected in each of the three orthogonal views (6 planes). Then, the images were cropped by these boxes, and the cropped images were used in the entire work flow (see Fig. 2).

To suppress the noise and increase the voxel intensity homogeneity within the liver, which is helpful for image registration and segmentation, the CT and CBCT images were filtered by three consecutive 2D median filtering with a width of 3 by 3 voxels in all three dimensions. This filter better preserves the organ’s edges than Gaussian filtering (see Fig. 2). The smoothed images were used as an intermediate for segmentation and registration, and the unsmoothed images were also processed in each step.

#### Image registration

The aim is to have all images in an identical space, and we refer to the resulting set of aligned images as the meta-image. Image registration was done in two steps:

First, early and late CBCTs for each LPT were aligned by a rigid registration. This multi-resolution registration used the sum of square differences as the cost function. After registration, averaging over all CBCTs creates an image which covers a larger field of view than the individual CBCTs, because each image is typically focused on a single liver lobe. We refer to this image as meta-CBCT. In addition, the contrast enhancement is suppressed due to averaging which helps further registration.

Then, the meta-CBCT and CT from the ^18^F-FDG study (CT_FDG_) images were registered to the CT from the MAA study (CT_MAA_) non-rigidly (starting from a initial rigid registration), and the PET image was deformed with the deformation obtained for the corresponding CT image. CBCT images were acquired in breath hold. ^18^F-FDG-PET/CT and MAA SPECT/CT imaging allowed shallow breathing. Consequently, breathing impact is unavoidable [74]. These images were acquired in clinical routine and were not gated for respiratory movement. So, in this study, breathing motion is part of the uncertainties.

The multi-resolution non-rigid registration was done with the algorithm described in [75]. Mutual information was used as the cost function. This algorithm represented the deformation with a displacement vector in every voxel. The deformation was constrained by assuming that neighboring voxels are connected by nonlinear springs, which can have different rigidity for voxels belonging to different classes. The image was segmented by thresholding into the air, liver, other soft tissues, and bone. The rigidity of the liver was set to a relatively high value, while that of all other tissues was set to a low value. This was done to ensure that the liver registration is good and as rigid as possible and that it is not hindered by alignment of the other structures surrounding the liver. Moreover, the registration is mostly driven by the high-intensity gradients near the liver boundaries. By favoring rigidity, a useful alignment for structures inside the liver can be obtained. Indeed, the perfusion and ^18^F-FDG uptake will not always be matched, and identifying mismatches is highly relevant for treatment planning/verification (see Fig. 1). The CBCTs and CT_MAA_ acquisitions were within hours from each other with minimal organ change in between.

#### Evaluation of registration methodology

##### Expert evaluation:

To evaluate CT_FDG_ to CT_MAA_ non-rigid registration, an expert (CMD) scored the results by using a 5-point Likert method. The co-registered datasets were displayed in an orthogonal viewer with transverse, coronal, and sagittal sections and were scored by using these predefined categories:

1. Major misalignment; major impact on dosimetry, dosimetry results not reliable
2. Pronounced misalignment; substantial impact on dosimetry is expected
3. Moderate misalignment; little impact on dosimetry is expected
4. Little misalignment; no significant impact on dosimetry is expected
5. Near perfect alignment; no intervention warranted and dosimetry deemed reliable

The following positions were inspected using a cursor point consisting of intersecting orthogonal lines:

- Cranial, caudal, lateral, and medial most extreme positions of the liver contour
- The hilar fat at the portal bifurcation
- The ligamentum falciforme
- The gallbladder bed
- Inlying calcifications in the liver (most often due to calcified liver metastasis), if present
- Liver metastasis if visible on the non-contrast-enhanced CT
- Biliary cysts, if present
- Coils in hilar vascular structures from pre-SIRT work-up, if present

##### Local volume change:

The liver is assumed to be a non-compressible organ. One can consider the healthy tissue/tumor volume changes over time owing to the time difference between the ^18^F-FDG and ^99m^Tc-MAA study, but still we assumed that local volume changes should be small and could be used as a metric in the evaluation of the registration results.

We used the Jacobian determinant [76, 77] to evaluate to which extent CT_FDG_ to CT_MAA_ registration was locally volume preserving in three different volumes of interest (VOIs) for each patient: the entire liver, excluded liver, and eroded liver. For the entire liver, the Jacobian of the voxels belonging to the manual liver segmentation was analyzed. For the eroded liver, the manual liver segmentation eroded by a 10-mm kernel was used and the excluded liver was defined as the entire liver subtracted by the eroded liver.

The most obvious feature of the liver used by the registration algorithm is its boundary, in particular for non-contrast-enhanced CT images. The Jacobian is computed in a region near the liver boundary to evaluate the balance between rigidity and similarity (“excluded liver”). In non-contrast-enhanced CT images, the center of the liver contains very few features, and the deformation in the center is mostly driven by the deformation of the boundaries and the rigidity constraint. To evaluate the propagation of the deformation to the center of the liver, the Jacobian is also computed in the central part (“eroded liver”).

#### Image segmentation

To define all the VOIs needed for the dosimetry report, one needs to delineate the entire liver, the tumors, and the different liver perfusion territories. All the variables which were used for the liver, tumor, and LPT segmentation and validation are listed in Table 1.

**Table 1:** List of variables that are used in VOI segmentation and validation

Step	Variable	Description
Tumor segmentation	THR (init)	Initial threshold of FDG uptake for tumor core definition
Tumor segmentation	BG (*T*_*i*_)	Measured background of tumor *i*
Tumor segmentation	max (*T*_*i*_)	Measured maximum FDG uptake of tumor *i*
Tumor segmentation	THR (*T*_*i*_)	Final threshold for tumor *i*
Liver evaluation	DICE	Dice coefficient
Liver evaluation	TPR	True positive ratio (sensitivity)
Liver evaluation	PPV	Positive prediciton value
Liver evaluation	RV	Relative volume
Liver/LPT evaluation	aHD	Average Hausdorff distance
Liver/LPT evaluation	mHD	Maximum Hausdorff distance
LPT evaluation	Vdiff	Volume difference
LPT evaluation	Rratio, Lratio	Right and left volume ratio

**Fig. 3:**
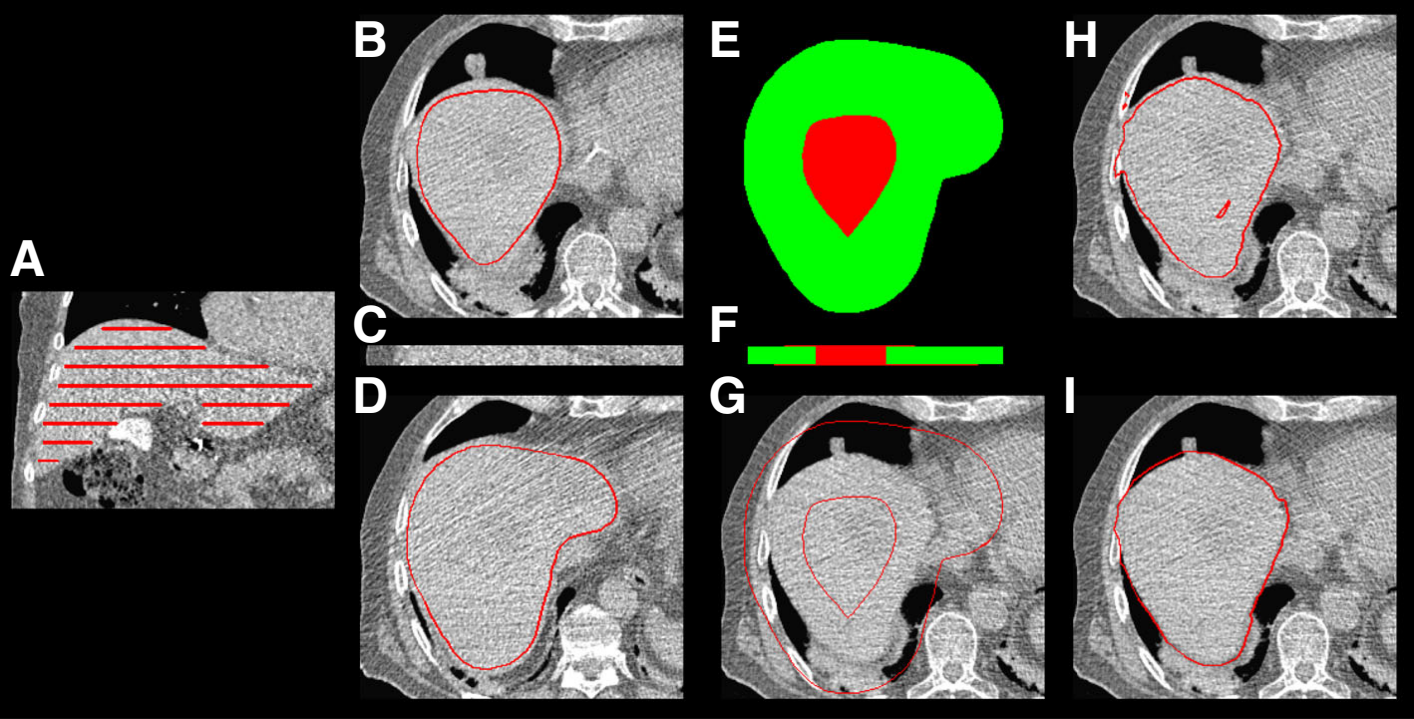
Semi-automatic liver segmentation steps (patient ID BI01 from training set in our database), **a** manual liver segmentation in every 15th slices, **c** a layer and **b**, **d** transverse slice of the upper and the lower plane of a sample layer and their manual segmentation, **e**, **f** the layer region growing seed (red) and mask (green), **g** a slice in the middle of the layer and its seed and mask, **h** initial region growing result, and **i** final segmentation

**Liver segmentation:** The entire liver was segmented by a joint region growing by using information from three co-registered images at the same time (CT_MAA_, CT_FDG_, and the ^18^F-FDG PET). This procedure benefits from co-segmentation; the liver is segmented based on the low-dose and low-quality CT from MAA study which is the most important pre-treatment study, but information from ^18^F-FDG study also helps the algorithm to have better initial liver segmentation and smoother manual modification by the expert. Details about liver segmentation are provided as supplementary material (Fig. 3).

**Tumor segmentation:** An adaptive thresholding method was used for tumor segmentation. This method is very similar to the fixed threshold level, but the threshold level is calculated from the tumor-specific background and tumor max SUV [78, 79]. First, the ^18^F-FDG PET image was converted to SUV values. Then, a mean (*μ*) and standard deviation (*σ*) were computed within the liver. To find the tumor cores, an initial threshold (THR(init)) was set to (confidence interval of 99.5% corresponding to 2.802 sigmas):

1 $$ \text{THR(init)}=\mu + 2.802\times{\sigma}  $$

**Fig. 4:**
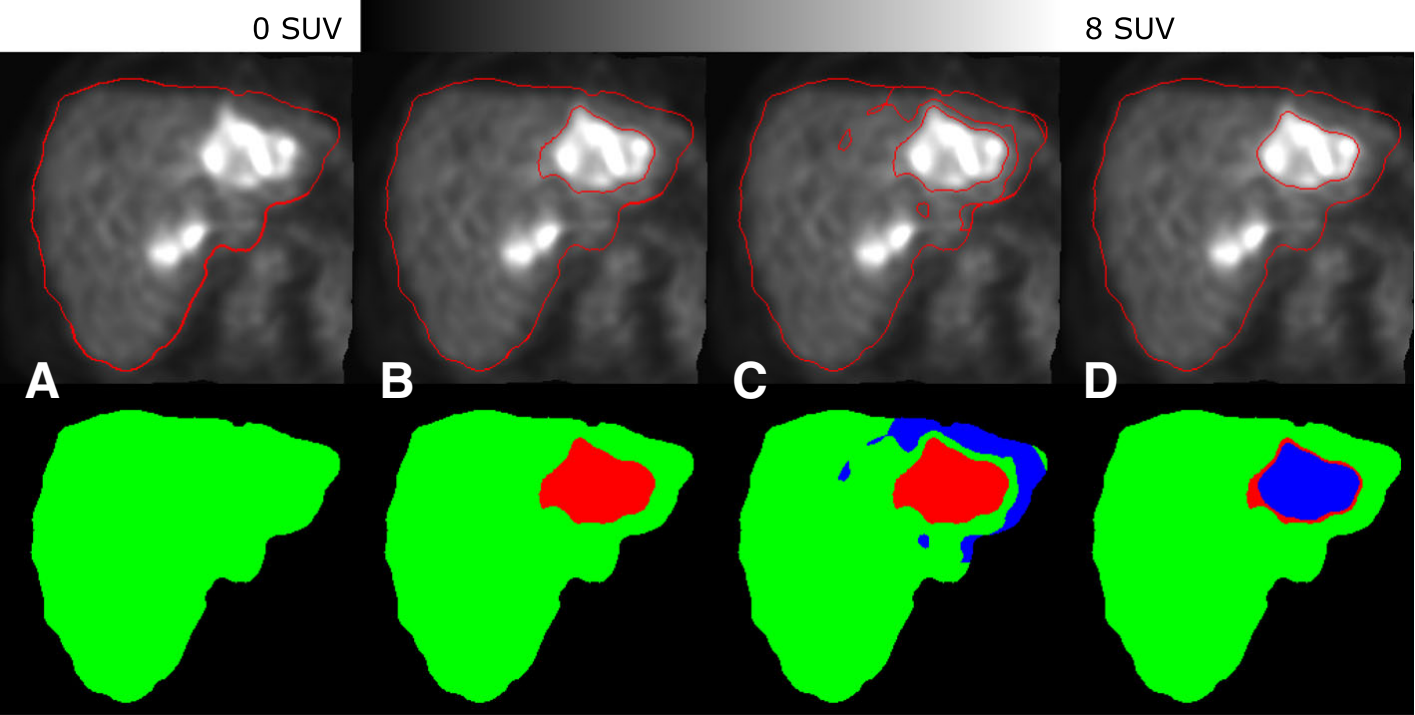
Tumor segmentation steps for one of the tumors (patient ID BI01 from training set in our database) that is visible in the slice by using the method proposed in [78]. For the other tumor(s), the same algorithm was used **a** initial ^18^F-FDG PET and (liver segmentation: green), **b**^18^F-FDG initial threshold and finding initial tumor burden (liver segmentation, green ; tumor cores, red), **c** finding specific background for each tumor (liver segmentation, green; tumor cores, red; background, blue), and **d** final tumor thresholding (liver segmentation, green; initial tumor volume which is not included in the final tumor segmentation, red; final tumor segmentation, blue)

Then, each of the detected tumor cores was independently processed to yield a final tumor volume. A mask was generated for each tumor core (*T*_*i*_) after dilation with a uniform sphere of radius 25 mm. The background for *T*_*i*_ (BG(*T*_*i*_)) was defined as the mean value of all voxels in the mask that had an SUV of less than 2.5, the tumor-specific threshold (THR(*T*_*i*_)) was defined as [78] (see Fig. 4):

2 $$ \text{THR}(T_{i})=\text{BG}(T_{i})+0.41\times{(\text{max}(T_{i})-\text{BG}(T_{i}))}  $$

**Fig. 5:**
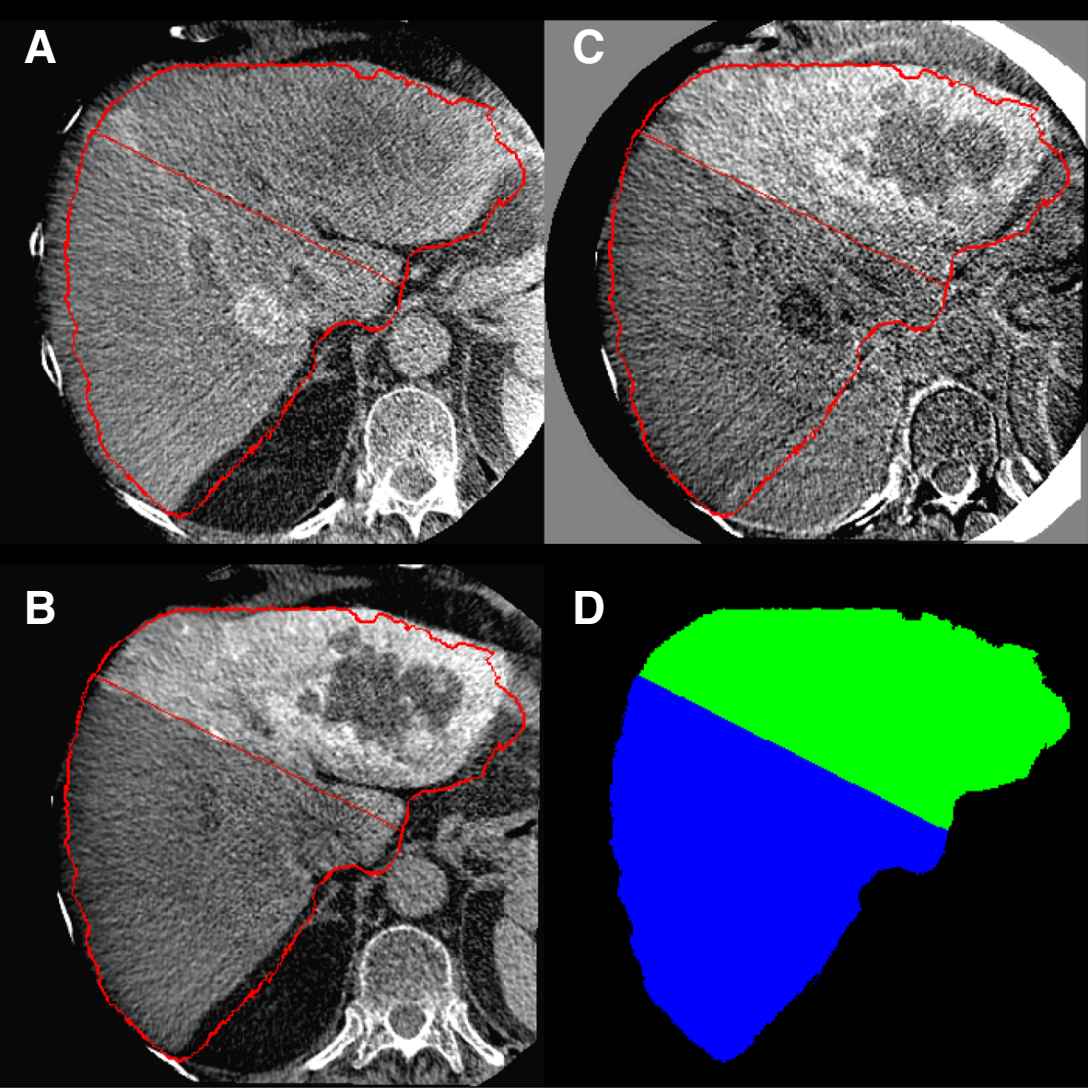
Perfusion territory segmentation (patient ID BI01 from training set in our database): **a**, **b** CBCTs with late contrast enhancement for the right and left liver perfusion territory, respectively, **c** difference between two CBCTs, **d** LPT segmentation result (right lobe, blue; left lobe, green)

**Perfusion territory segmentation:** The task is to separate the left and right LPT based on the contrast enhancement in the early or late arterial phase of the CBCT. To segment the liver in two lobes, an expert drew lines in at least 3 different transverse slices, then a plane was fit to these lines by using the least square method. The plane was reviewed by the same expert in all 4 sets of CBCTs and revised when needed (see Fig. 5).

#### Liver segmentation comparison and validation

A second segmentation of the liver by an expert (WC) on a single contrast-enhanced CT was used to validate our segmentation methodology. This manual segmentation was done using Siemens Syngo MMWP Volume software [80].

Our segmentation results were compared to those obtained by manual segmentation using dice coefficient, true positive ratio (TPR) or sensitivity, positive predictive value (PPV), relative volume (RV), average Hausdorff distance (aHD), and maximum Hausdorff distance (mHD). Dice coefficient is the most used method to evaluate volume overlapping, TPR shows the ratio of the gold standard that is covered by our segmentation, PPV shows the fraction of our segmentation that is covered by the gold standard, RV reports the volume ratios, and mHD and aHD are based on distances between the boundaries of the two segmentations [81, 82].

Dice index, TPR, PPV, RV, aHD, and mHD between a volume A and the gold standard segmentation B are given by:

3 $$ {\begin{aligned}  \left\{ \begin{array}{l} \text{DICE}= \frac{2\times{|A \bigcap B|}}{|A|+|B|} \\ \text{TPR}=\frac{\textrm{Number of true positives}} {\textrm{Volume of the gold standard}} = \frac{|A \bigcap B|}{|B|}\\ \text{PPV}=\frac{\textrm{Number of true positives}} {\textrm{Volume of the test}} = \frac{|A \bigcap B|}{|A|}\\ \text{RV}=\frac{\textrm{Volume of the test}} {\textrm{Volume of the gold standard}} = \frac{|A|}{|B|}\\ \text{aHD[mm]}=\frac{1}{|S(A)|+|S(B)|}\times{\left(\sum\limits_{s_{a} \in S(A)} \min\limits_{s_{b} \in S(B)} {\| b-a \|} +\sum\limits_{s_{b} \in s(B)} \min\limits_{s_{a} \in S(a)} {\| a-b \|} \right)} \\ \text{mHD[mm]}=\max {\left[ \max\limits_{s_{a} \in S(A)} {\min\limits_{s_{b} \in S(B)} {\| b-a \|} }, \max\limits_{s_{b} \in S(B)} {\min\limits_{s_{a} \in S(A)} {\| a-b \|}} \right] } \end{array}\right. \end{aligned}}  $$

Here, S(X) denotes the set of surface voxels of X and ∥.∥ is the Euclidean distance in mm.

#### Impact of using the CBCT images instead of the CT image for defining LPT

To evaluate our methodology to delineate LPTs of different branches of the hepatic artery (left and right hepatic artery in our patient selection), a second segmentation was done on a single CT by defining a plane to cut the CT for segmenting two different LPTs by using the anatomical landmarks. To manually segment the liver in two lobes, the expert (WC; who performed lobe segmentation on CBCT) segmented the LPTs on the CT images with the same tool that has been used to segment them on the CBCT images. To avoid bias, the expert segmented the LPTs on CT 2 weeks after CBCT-based LPT segmentation. Then, this separation plane was used to define different LPTs by using the transformed liver segmentation.

The VOI for the left and right LPT were compared by using volume difference (Vdiff), left LPT volume ratio (Lratio), right LPT volume ratio (Rratio), aHD, and mHD.

Left/right volume ratio was used to compare the left/right volumes derived from these two LPT segmentations; these parameters can be re-formulated as the ratio of RtoW and LtoW, which calculate right and left LPT to entire liver volume. LtoW and RtoW are two key parameters in dosimetry and therapy planning in SIRT. They are used in most of the activity calculation methods (BSA, partition method) to split total activity into the right and left lobe activity:

4 $$ \left\{\begin{array}{l}  \text{RtoW} = \frac{\textrm{Volume of the right LPT}}{\textrm{Volume of the entire liver}} \\ \text{LtoW} = \frac{\textrm{Volume of the left LPT}}{\textrm{Volume of the entire liver}} \end{array}\right.  $$

Volume difference is the liver volume which is assigned to different lobes by CBCT and standard venous anatomical lobe segmentation, normalized by total liver volume. Similar to the liver segmentation evaluation, aHD and mHD are based on distances between the (operator-defined) planes that are used to separate the LPTs.

5 $$ {\begin{aligned} \left\{\begin{array}{l}  \text{Vdiff}= \frac{|L_{A} \bigcap R_{B}|+|L_{B} \bigcap R_{A}|}{|L_{B} \bigcup R_{B}|} \\ \text{Rratio}= \frac{|R_{B}|}{|R_{A}|} = \frac{\textrm{RtoW of the CT-based lobe segmentation}}{\textrm{RtoW of the CBCT-based lobe segmentation}}\\ \text{Lratio} =\frac{|L_{B}|}{|L_{A}|}=\frac{\textrm{LtoW of the CT-based lobe segmentation}}{\textrm{LtoW of the CBCT-based lobe segmentation} }\\ \text{aHD[mm]}=\frac{1}{|S(A)|+|S(B)|}\times{\left(\sum\limits_{s_{a} \in S(A)} \min\limits_{s_{b} \in S(B)} {\| b-a \|} +\sum\limits_{s_{b} \in s(B)} \min\limits_{s_{a} \in S(a)} {\| a-b \|} \right)} \\ \text{mHD[mm]}=\max {\left[ \max\limits_{s_{a} \in S(A)} {\min\limits_{s_{b} \in S(B)} {\| b-a \|} }, \max\limits_{s_{b} \in S(B)} {\min\limits_{s_{a} \in S(A)} {\| a-b \|}} \right] } \end{array}\right. \end{aligned}}  $$

where *L*_*A*_ and *R*_*A*_ are the left and right LPT volumes defined by the expert on CBCT images, respectively, and *L*_*B*_ and *R*_*B*_ are the left and right LPT volumes defined on the CT images. S(A) denotes the set of voxels in the segmented liver which lie on the plane which separates the left and right LPTs. The LPT segmentation in S(A) was derived from the the contrast CBCT-based segmentation. S(B) is defined in the same way for lobe segmentation based on anatomy (on CT).

#### Role of perfusion territory segmentation in dosimetry

In the SIRT workflow, the volumes of the right and left LPTs are used to compute the activities that should be administered to each of the lobes [13, 70, 71]. Our hypothesis is that CBCT contains more accurate information about perfusion territories than CT which only contains anatomical landmarks to distinguish the left and right liver lobes and is based on the venous anatomy (which sometimes is different from the arterial perfusion territories). To estimate the impact of using standard venous anatomical lobe segmentation on CT instead of LPT segmentation on CBCT, a simulation was performed:

First, the injected activity for each LPT was calculated by using the non-compartmental partition method [83] and lobe segmentations based on anatomical landmarks (current clinical workflow):

6 $$ {\begin{aligned} \text{IA}_{\text{partition}} (\text{LPT}) [\text{GBq}] = \frac{D_{\text{liver}}[\text{Gy}]\times{\text{CT-based lobe mass [kg]} }}{49.380\left[\nicefrac{\text{Gykg}}{\text{GBq}}\right]} \end{aligned}}  $$

where *D*_liver_ is the desired mean dose to the total liver and 40 Gy was used based on the algorithm by the Gil-Alzugaray et al. study [73]. In our calculation, lobe volume was converted to lobe mass by using 1.03g/c*m*^3^ as the liver density [83].

Then, a second partition dosimetry was performed to evaluate the absorbed dose for the corresponding injected activity in each LPT by using CBCT-based lobe segmentation results to calculate actual mean absorbed dose (unit: Gy) in each lobe by assuming CBCT-based lobe segmentation as the ground truth:

7 $$  D_{\text{LPT}} [Gy]= \frac{49.380 \left[\nicefrac{\text{Gykg}}{\text{GBq}}\right] \times{\text{IA}_{\text{partition}} (\text{LPT}) [\text{GBq}]}}{\text{CBCT-based lobe mass [kg]}}  $$

The impact of using CBCT instead of anatomical LPT segmentations is then assessed by computing the difference of *D*_LPT_ of Eq. 7 from the intended dose of 40 Gy.

## Results

### Evaluation of registration methodology

**Fig. 6:**
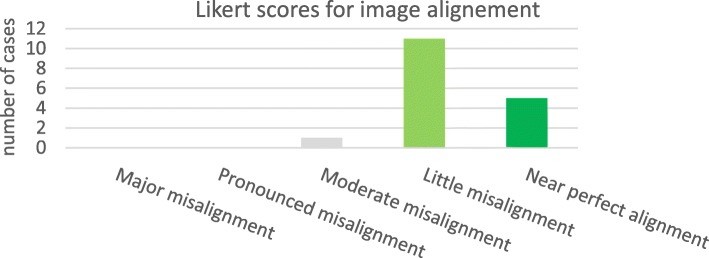
Expert Likert score for CT_FDG_ to CT_MAA_ registration

**Fig. 7:**
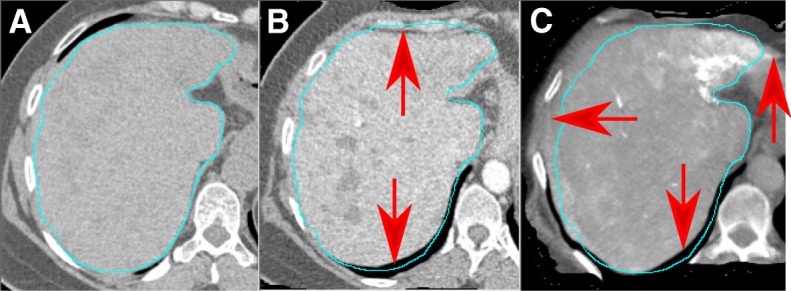
An example (patient ID BI02 from training set in our database) of mis-registration for CT_FDG_ and CBCT to CT_MAA_ non-rigid registration (Likert score for this patient is “little misalignment,” contours come from automatic liver segmentation and red arrows point to mis-registration area. **a** Reference image (CT_MAA_), **b** deformed CT_FDG_, **c** deformed meta-CBCTs

**Expert evaluation:** The registration of CT_FDG_ to CT_MAA_ was reviewed by the nuclear medicine expert (CMD). The results for the registration evaluation are shown in Fig. 6 and demonstrate that the proposed method for registering CT_FDG_ to CT_MAA_ was near perfect for 5 patients (29%) and with little misalignment for 11 patients (65%), with moderate misalignment for one patient (6%), and none of the cases were scored as “pronounced misalignment” or “major misalignment”. Figure 7 shows an example of little misalignment for CBCT and CT_FDG_ to MAA non-rigid registration.

**Fig. 8:**
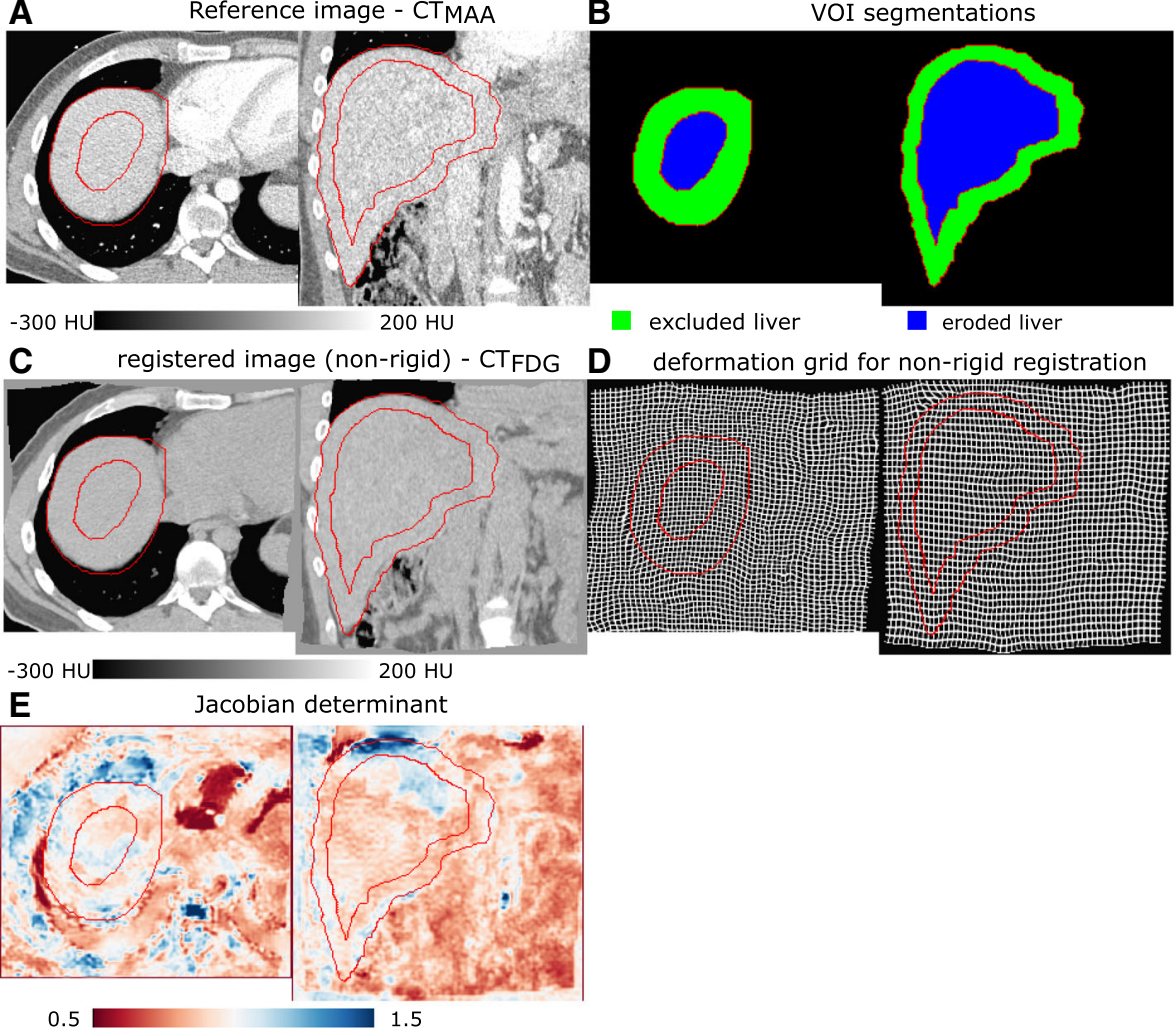
An example (patient ID BI04 from training set in our database) of transaxial and coronal view of non-rigid registration evaluation for a patient with Likert score of “little misalignment,” the statistics [min, Q1, median, Q3, maximim] of the Jacobian over all liver voxels, eroded liver, and excluded liver for this patient are [0.42, 0.82, 0.89, 0.97, 1.74], [0.58, 0.83, 0.88, 0.96, 1.45], and [0.42, 0.82, 0.90, 0.99, 1.74], respectively, (red contours are from the excluded liver and eroded liver—see **b**): **a** reference image CT_MAA_, **b** excluded and eroded liver: eroded liver is manual liver segmentation eroded by 10 mm and excluded liver is the subtraction of excluded liver from liver **c** and **d** results of non-rigid registration (CT_FDG_ to CT_MAA_) and the deformation grid and **e** Jacobian determinant of the non-rigid registration: blue, local dilation; red, local contraction; and white, no local volume change

**Local volume change:** The Jacobian determinant for non-rigid registration deformation is a parameter which shows the local volume change. A Jacobian determinant equal to 1 corresponds to no volume change, greater than 1 corresponds to a local dilation, and less than 1 corresponds to a local contraction. Negative Jacobian shows up where the deformation is locally non-revertible. Figure 8 shows a transaxial and coronal slice of the non-rigid registration with Jacobian determinant distribution for a patient.

**Fig. 9:**
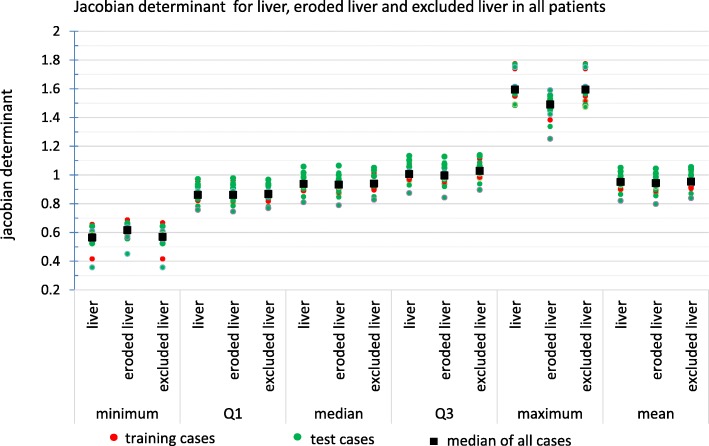
First, second, and third interquartile range, minimum, maximum, and mean of for Jacobian determinant for the liver, eroded liver (liver eroded with 10 mm), and liver excluded (subtraction of eroded liver of liver), this measure shows the local dilation (Jacobian greater than 1), local contraction (Jacobian less than 1), and no local volume change (Jacobian 1) for each voxel for liver voxels, core of the liver, and border of the liver

Figure 9 shows the statistics of the Jacobian determinant. The median of the Jacobian determinant of liver voxels ranged from 0.81 to 1.05 for all cases, while the median range for eroded liver and exclude liver voxels were very similar ([0.79,1.07] and [0.83,1.05] respectively). But higher local contraction occurred in excluded liver (minimum of Jacobian ranged from 0.36 to 0.67) compare to eroded liver by minimum Jacobian range of [0.45,0.69]. Also, the excluded liver had higher local dilation (maximum of Jacobian ranged from 1.47 to 1.78) compared to the eroded liver which had a maximum Jacobian range of [1.25,1.59].

### Liver segmentation comparison and validation

**Fig. 10:**
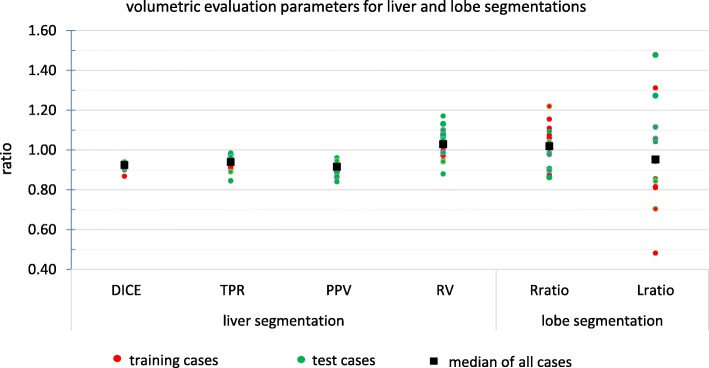
Volumetric evaluation of liver and lobe segmentation for training and test cases. Dice coefficient, true positive ratio, positive protective value, and relative volume for liver manual and automatic segmentation and ratio between right/left lobe volumes for two different lobe segmentation procedures (anatomical lobe segmentation on CT and LPT segmentation on CBCT)

Our semi-automatic liver segmentation used joint region growing by using information from CT_MAA_, CT_FDG_, and ^18^F-FDG PET. The results of the joint region growing were manually adjusted for all the patients. The initial liver segmentation (seed for region growing) took up to 4 min (median =3 min) and final adjustment took up to 6 min (median = 4 min). Figure 10 illustrates the performance of the total liver segmentation by 4 volumetric accuracy metrics (dice, TPR, PPV, and RV) for all training and test cases. Statistics of these metrics for all patients (test cases and training set) are summarized in Table 2. Median and mean of the dice coefficient were both 0.92 showing very similar liver segmentation by both methods with very narrow interquartile range. TPR and PPV had a median of 0.94 and 0.92 which means on average 94% of the liver, which was segmented with manual segmentation, was also segmented with automatic segmentation, and 92% of automatic segmented liver volume was also segmented by the manual segmentation. RV is a very important parameter for injected activity planning. If the partition method or BSA method is used for calculating injected activity, the volume of the liver plays an important role. So, the ratio between the volumes is a key parameter in liver segmentation for SIRT. Our analysis shows that the median (i.e., 1.03) was very close to 1. The accuracy of the segmentation is important if one wishes to use small scale dosimetry (e.g., voxel level dosimetry). The average (aHD) and maximum (mHD) distance between the liver surfaces obtained with the semi-automated method and by the expert were 3 and 22 mm, respectively.

**Table 2:** Evaluation of total liver segmentation for all cases (test set and training set were pooled together); dice coefficient, true positive ratio, positive protective value, relative volume, and maximum and average of Hausdorff distances for manual and automatic liver segmentation

	Dice	TPR	PPV	RV	mHD (mm)	aHD (mm)
Minimum	0.87	0.85	0.84	0.88	16.93	2.09
Q1	0.91	0.91	0.89	0.98	20.54	2.51
Median (Q2)	0.92	0.94	0.92	1.03	22.39	3.04
Q3	0.93	0.96	0.94	1.08	30.62	3.16
Maximum	0.94	0.99	0.96	1.17	47.26	4.23
Average	0.92	0.94	0.92	1.03	22.39	3.04

#### Impact of using the CBCT images instead of the CT image for defining LPT

**Fig. 11:**
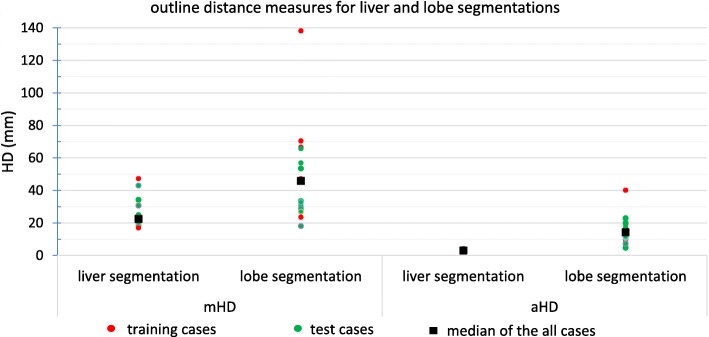
Outline maximum and average Hausdorff distance measures (mHD and aHD) between manual and automatic liver segmentation and two different lobe separation planes (on CT and on CBCT) for training and test cases

**Fig. 12:**
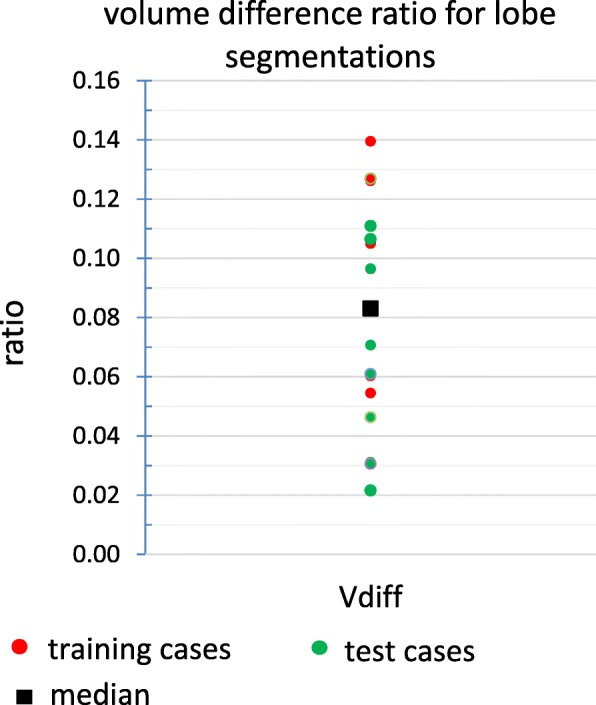
Volume difference parameter. The liver volume which was assigned to different lobes by standard venous anatomical (using CT) and perfusion (on CBCT) lobe segmentation for both training and test cases

**Fig. 13:**
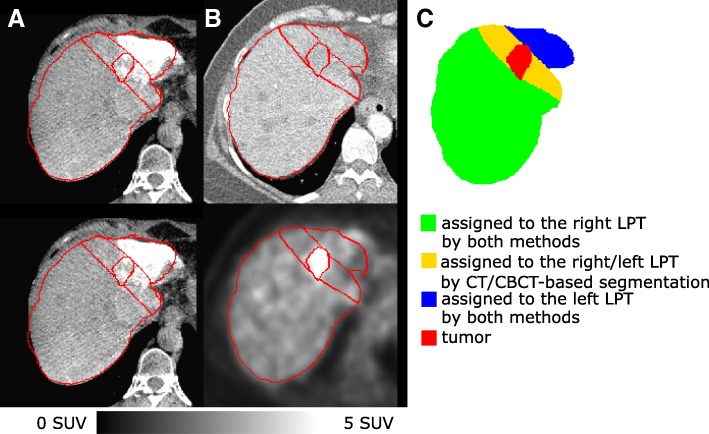
An example (patient ID BI02 from training set in our database) of **a** transaxial view of early and late CBCTs, **b** the ^18^F-FDG PET/CT images, as well as, **c** CBCT/CT-based LPT segmentation and tumor segmentation (from ^18^F-FDG PET). In this slice, the tumor was segmented in different lobes by the two methods. Comparing the CBCT- and CT-based lobe segmentation for this patient showed that 10% of the liver volume was assigned to different lobes (Vdiff), the ratio of “right lobe to entire liver volume ratio” for these two methods (Rratio) was 1.08 and the same parameter for the left lobe (Lratio) was 0.82. Furthermore, the average and maximum distance between lobe separation planes by these two methods (aHD and mHD) were 2.74 and 16.94 mm. Dosimetry simulation showed that a mean absorbed dose to the left and right lobe when using CBCT-based lobe segmentation were 19 and 46 Gy respectively, when we aimed at delivering 40 Gy to each lobe using the CT-based LPT segmentation

Results for the volumetric comparison of the two different lobe segmentation procedures for the test and training sets are illustrated in Figs. 10, 11, and 12, more detailed results for all 17 cases (10 test and 7 training cases) can be found in Table 3. Figure 13 shows an example with a large area of disagreement between CBCT- and CT-based LPT segmentation. In this particular case, a tumor was segmented in different LPTs by each of the two LPT segmentation methods.

**Table 3:** Evaluation of lobe segmentation for all cases (test set and training set); volume difference, right lobe volume ratio, left lobe volume ratio, and maximum and average of Hausdorff distance for CT- and CBCT-based lobe segmentation

	Vdiff	Rratio	Lratio	mHD (mm)	aHD (mm)
Minimum	0.02	0.86	0.48	18.01	4.69
Q1	0.05	0.98	0.84	30.13	10.31
Median (Q2)	0.08	1.02	0.95	45.80	14.18
Q3	0.11	1.08	1.06	56.91	16.97
Maximum	0.14	1.22	1.48	138.16	40.08
Average	0.08	1.02	0.97	48.00	15.10

Surface distances are also provided in Table 3 and Fig. 11.

Median of Vdiff (area of disagreement for lobe segmentation) was 8% of the liver volume, and in some cases, it was as high as 14%. This is an important source of error for high level dosimetry (e.g., compartmental partition model and voxel level dosimetry) when there is a tumor in this disagreement area (see Fig. 13).

The median of the ratio between left/right lobe ratio index that has a direct effect on splitting the total prescribed injected activity in all prescription methods was near 1 (1.02 and 0.95 for Rratio and Lratio, respectively), but the deviation from 1 was very high, and for some cases, this deviation was up to 48% (see “Role of perfusion territory segmentation in dosimetry” section).

The median of aHD and mHD between the two separating planes for the all patients were 3.04 and 22.39 mm, respectively. This shows a possible error for compartmental partition model and voxel level dosimetry because the same tumor can be assigned to a different liver perfusion territory, depending on the procedure used to segment the liver lobes. This would be very problematic for using the dosimetric result either for finding injected activity or post-treatment verification (see Fig. 13).

### Role of perfusion territory segmentation in dosimetry

**Fig. 14:**
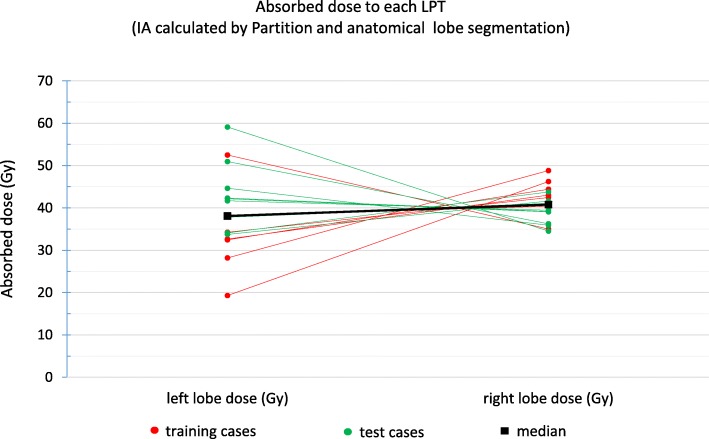
Dosimetry simulation result in each lobe when we aimed at 40 Gy for each lobe based on standard venous anatomy delineation and using CBCT-based LPT segmentation and injected activity calculated by the partition method

For analyzing the effect of using the standard venous anatomical lobe definitions instead of the perfusion territories, a simulation was designed to calculate the absorbed dose in each CBCT-based LPT. Our dosimetry calculations showed that when we aim at delivering 40 Gy to the total liver, a median absorbed dose to the right CBCT-based LPT (using the standard venous anatomical lobe segmentation) was 40.8 with a [min, max] deviation of [–5.9, 8.8] Gy from 40 Gy. For the left lobe, the deviation was wider, and the median absorbed dose was 38.1 Gy; however, the range of deviation was [–20.7, 19.1] Gy. Figure 14 shows the results of the absorbed dose in the left and right LPTs (Fig. 13 shows a transaxial view slice of a LPT segmentations).

## Discussion

Our aim was the development of a semi-automatic procedure for registration and segmentation of all the images acquired in the planning of and during SIRT The tool aligns all available images and provides semi-automatic delineation of the total liver, the liver perfusion territories, and the tumour lesions. The total liver is delineated on PET/CT, the perfusion territories on CBCT, and the tumours on ^18^F-FDG PET/CT. Since no ground truth is available, we have evaluated the semi-automated segmentations by comparison to manual expert segmentations and the registrations were evaluated visually by an expert.

### Validation of registration

Because in our method, we used information from all images to segment different VOIs, the accuracy of registration plays an important role. Also the alignment between the ^99m^Tc-MAA SPECT/CT images and the final segmentation highly affect the dose volume histograms and dosimetry analysis.

The assumption is that in our registration algorithm, the deformations inside the liver were more driven by the liver boundary registration than by correct alignment of inner liver structures. For that reason, we maximized the rigidity inside the liver, in the hypothesis that this better approximates the true deformation.

Maximizing the rigidity of non-rigid registration, also, favors local volume preservation in liver voxels. A Jacobian determinant was used to estimate this local volume change due to non-rigid registration. The result of Jacobian determinant analysis showed relatively small volume change for central liver voxels. Bigger volume changes (either contraction or dilation) were observed near the border of the liver.

Sometimes these assumptions (high rigidity of liver movement and lack of local volume change of the liver) are questionable because of some small non-rigid deformation of the liver and because of anatomical liver changes over time, but still it seems reasonable to limit the non-rigidity of liver registration. In those cases, our parameter selection may contribute to mis-registration errors. Another possible source of error in registration could be the artifacts in the CT images (e.g., streak artifacts or photon starvation due to elbows inside field-of-view) and non-rigid liver deformation due to patient position. Likert scoring results for registration accuracy showed that this mis-registration was not crucial in most of the cases of CT_FDG_ to CT_MAA_ registration.

In clinical routine, the Jacobian can be used as a quality control step to warn physicians about possible registration problems. Our visual investigation showed that the upper and lower part of the liver were the areas with deviation from Jacobian determinant of 1 for most of the cases. In general, the results suggest that the registration error was low compared to other sources of error in SIRT dosimetry, but in areas with mismatch between ^18^F-FDG PET and ^99m^Tc-MAA SPECT, we suggest to look more carefully into registration deformation to avoid underestimation of tumor dose/activity and overestimation of healthy tissue dose/activity.

One of the limitations of this study is that during MAA SPECT/CT and ^18^F-FDG-PET/CT, shallow breathing was allowed and breathing motion is not corrected. It has been shown that respiratory motion can impose a large error into final dosimetry results. Bastiaannet et al. report 90 to 66% decrease in activity recovery and tumor-to-normal ratios because of respiratory motion in a series of SPECT/CT Monte Carlo simulation using digital XCAT phantoms [74].

### Validation of liver segmentation

Liver segmentation is very important for dosimetry-based prescription of activity. Dice, TPR, and PPV parameters showed a high similarity between our semi-automatic segmentation results and manual liver segmentation by an expert, which is used in clinical routine. A comparison between our liver segmentation results and the accurate methods on liver segmentation reviewed by Moghbel et al. [40] shows that our Dice metric (mean = 0.92) is similar to what is reported in previous published liver segmentation algorithms ([0.91,0.94]), and our relative volume metric (mean =1.03) is comparable with the result of the most accurate algorithms ($[0.943,0.983]\bigcup [1.003,1.075]$).

These results show that our semi-automatic liver segmentation was comparable with manual liver segmentation by an expert. Because this segmentation method is applied to a multi-modal image (PET and CT), it uses more information, and therefore, its segmentations could even be slightly superior. This will be further investigated in the future.

### Validation of the lobe segmentation

In standard clinical routine, the lobe segmentation is done on a CT image based on anatomical landmarks, predominately based on venous structures. Here, we studied an alternative approach, where the lobes were segmented on contrast-enhanced CBCT images. A graphical user interface was developed to cut the liver on CBCT and CT by drawing just a few separation lines on different transverse slices, it usually takes less than a minute for the expert for each case.

In several cases, the expert separated the liver differently into lobes, depending on whether anatomical CT images or perfusion CBCT images were being used. Evaluation of these two different lobe segmentation procedures suggested that there was a relatively large area in the center of the liver where the standard venous anatomical lobe segmentation on CT differed from the perfusion-based segmentation on CBCT. This can cause a large error for total administered activity distribution in bilobar treatment and in dosimetry. If there is a tumor in this disagreement area, there is a risk of assigning the tumor to the wrong perfusion territory, which could lead to serious underdosing of the tumor (see Fig. 13).

### Contribution of lobe segmentation methodology in dosimetry uncertainty

LtoW and RtoW—ratio between left/right to entire liver volume—(which is very important for dividing the total injected activity for left and right lobe) had a median of 0.95 (range [0.48, 1.48]) and 1.02 (range [0.86, 1.22]), respectively. The volume ratio of the left LPT to the entire liver had a wider range than right to entire because the left lobe is generally smaller than the right lobe and is more sensitive to mis-segmentation. It showed that the left lobe suffered more from under/over-treatment or under/over-irradiation by using the standard venous anatomical lobe segmentation as an estimate of the LPT. In our dosimetric setting, this under-treatment could be up to 21 Gy for the left lobe and 5 Gy for the right lobe. On the other hand, over-irradiation of the left lobe was up to 19 and 9 Gy for the left and right lobe, respectively.

### Tumour segmentation

In this study, we have focused on ^18^F-FDG-avid tumors that can be delineated by thresholding the ^18^F-FDG PET images. In this study, an adaptive threshold method was used for tumor segmentation which uses a tumor-specific threshold level by considering the SUV_max_ and background SUV for each tumor separately. Because thresholding the ^18^F-FDG-images is straightforward and the same as the method used by experts to delineate the lesions, we have not attempted to further evaluate the tumor delineation.

## Conclusion

A multi-modal image analysis approach was developed to obtain a personalized liver map (liver, perfusion territories, and tumor segmentation) from the pre-treatment ^99m^Tc-MAA SPECT/CT, ^18^F-FDG PET/CT, and CBCT images. The analysis showed that liver segmentation was comparable with manual segmentation by an expert. Liver perfusion territory segmentation by using CBCT instead of using CT and anatomical features showed to improve segmentation; the results showed a relatively high difference in volumetric parameters that are very important for SIRT in clinical routine.

Our results were based on 7 cases for optimizing and 10 test cases. Extending the number of patients can help us to evaluate our routine in clinical practice.

This procedure can be used in the future to provide semi-automatic voxel level fractional uptake predictions based on the ^99m^Tc-MAA SPECT/CT. By using this workflow, various dosimetry reports can be computed which can be used to evaluate and improve the safety and effectiveness of SIRT. This method can be used in the future for finding the injected activity more accurately by using either dosimetric methods (partition, voxel level dosimetry) or non-dosimetric methods (BSA, SIRFLOX look-up table [84]).

This procedure was designed for patients with ^18^F-FDG-avid tumors, but in the future, it can be extended to using magnetic resonance (MR) images instead of PET/CT images in patients with non ^18^F-FDG-avid tumors such as hepatocellular carcinoma.

## Appendix: Patient information

**Table 4:** Patient characteristics

Characteristics	All cases	Training set	Test set
Patient information			
Sex (female/male)	6/11	3/4	3/7
Age in years, median [range]	60.5 [32.9, 72.3]	60.6 [32.9, 72.3]	58.2 [36.2, 69.5]
Height in meter, median [range]	175 [160, 193]	174 [163, 186]	178 [160, 193]
Weight in kg, median [range]	81 [61, 123]	79 [71, 100]	84 [61,123]
Tumor cell type, n (%)			
Colorectal cancer	7 (41%)	1 (14%)	6 (60%)
Breast cancer	2 (12%)	2 (29%)	0 (0%)
Melanoma cancer	2 (12%)	2 (29%)	0 (0%)
Esophageal cancer	2 (12%)	1 (14%)	1 (10%)
HCC	1 (6%)	1 (14%)	0 (0%)
Neuroendocrine tumor	1 (6%)	0 (0%)	1 (10%)
Colon cancer	1 (6%)	0 (0%)	1 (10%)
Cholangiocellular	1 (6%)	0 (0%)	1 (10%)
Volumes from prescription sheet (cc), median [range]			
Total liver (WL)	1892 [1268, 3127]	1878 [1663, 2721]	1907 [1268, 3127]
Total liver (L-LPT)	448 [215, 1197]	390 [251, 1050]	514 [215, 1197]
Total liver (R-LPT)	1496 [677, 1930]	1330 [1223, 1839]	1533 [677, 1930]
Healthy liver (WL)	1700 [1218, 2988]	1700 [1513, 2528]	1694 [1218, 2988]
Healthy liver (L-LPT)	390 [135, 1115]	390 [201, 1041]	395 [135, 1115]
Healthy liver (R-LPT)	1319 [700, 1873]	1241 [1184, 1612]	1344 [700, 1873]
Tumor (WL)	150 [7, 643]	150 [28, 494]	153 [7, 643]
Tumor (L-LPT)	36 [0, 312]	18 [0, 109]	51 [0, 312]
Tumor (R-LPT)	130 [7, 479]	146 [10, 479]	93 [7, 331]
Clinical information from prescription sheet (%), median [range]			
Tumor involvement (WL)	7.1 [0.4, 27.8]	8.5 [1.5, 24.2]	5.7 [0.4, 27.8]
Tumor involvement (L-LPT)	6.9 [0.0, 47.3]	4.8 [0.0, 28.7]	8.7 [0.0, 47.3]
Tumor involvement (R-LPT)	8.6 [0.8, 27.7]	11.0 [0.8, 27.7]	6.5 [1.1, 20.0]
L-LPT to WL volume	25 [11, 60]	23 [12, 39]	27 [11, 60]
Lung shunt fraction (LSF)	6.4 [3.8, 8.5]	7.4 [3.8, 8.2]	5.8 [3.9, 8.5]
Day differences between different studies and SIRT (days), median [range]			
^18^F-FDG and SIRT	20 [3, 58]	35 [3, 58]	16 [6, 40]
^99m^Tc-MAA and SIRT	21 [15, 41]	21 [15, 32]	21 [15, 41]
Prescription			
IA calculation method			
BSA method, n (%)	14 (82%)	7 (100%)	7 (70%)
Partition model, n (%)	3 (18%)	0 (0%)	3 (30%)
IA in GBq, median [range]			
WL	1.850 [1.268, 2.502]	1.837 [1.746, 2.063]	1.882 [1.268, 2.502]
L-LPT	0.457 [0.228, 1.089]	0.457 [0.228, 0.724]	0.469 [0.248, 1.089]
R-LPT	1.339 [0.733, 1.783]	1.339 [1.113, 1.640]	1.414 [1.113, 1.640]

*WL* whole liver

*L-LPT* left liver perfusion territory

*R-LPT* right liver perfusion territory

## Supplementary material: Joint region growing for liver segmentation

A joint region growing method has been designed to delineate the liver using CT from MAA SPECT/CT, and CT and PET from ^18^F-FDG PET/CT study. The algorithm consisted of the following three steps:

- Initial liver segmentation: First, we manually segmented the liver in every 15th slice corresponding to 15 mm in the axial direction. The final liver segmentation was done independently in each of these volumes (of 15 slices) by the joint region growing algorithm. The eroded version (*r* = 20 voxels) of the intersection of two neighboring segmentations defined the seed for the joint region growing algorithm, and the dilated (*r* = 20 voxels) union determined the mask of the joint region growing algorithm. In the joint region growing algorithm, a voxel (*μ*_*j*_) within the selected mask was considered as the liver if its intensity satisfies:

8 $$ \forall i:\left|\mu_{j}-\mu^{i}\right| \le 2\times{\sigma^{i}},i=\text{CT}_{\text{MAA}},\text{CT}_{\text{FDG}},\text{PET}  $$

where *μ*^*i*^ and *σ*^*i*^ are the mean and standard deviation of the segmented voxels in image *i* in the previous step of region growing (see Fig. 3).

- Second region growing: The segmentation from the previous step was used as the seed for the second region growing. In this step, the dilated version of the resulting VOI by radius 20 voxels was used as the mask. In the second joint region growing algorithm, a voxel (*μ*_*j*_) within the selected mask was considered as the liver if its intensity satisfies:

9 $$ \forall i:k_{1}^{i}\times{\sigma^{i}} \le \mu_{j}-\mu^{i} \le k_{2}^{i}\times{\sigma^{i}},i=\text{CT}_{\text{MAA}},\text{CT}_{\text{FDG}},\text{PET}  $$

where *μ*^*i*^ and *σ*^*i*^ are the mean and standard deviation of the segmented voxels in image *i* in the previous step of region growing. $\left (k_{1}^{i},k_{2}^{i}\right)$ was set to (−2.5,2), (−2.5,2), and (−2,*∞*) for *i*=CT_MAA_,CT_FDG_,andPET, respectively, also *μ*_*j*_ was restricted to be below 2.5 standardized uptake value (SUV) for *i*=PET.

- Adjustment: Finally, the resulting VOIs were adjusted manually and closing and opening with 3 mm were applied.

## Abbreviations

^18^F-FDG: Fluorine-18 fluorodeoxyglucose
aHD: Average hausdorff distance
BSA: Body surface area
CBCT: Cone beam CT
CT: Computed tomography
HCC: Hepatocellular carcinoma
LPT: Liver perfusion territory
Lratio: Left LPT volume ratio
LtoW: Left LPT to entire liver volume
MAA: Macro-aggregated albumin
mCRC: Metastatic colorectal cancer
mHD: Maximum hausdorff distance
MR: Magnetic resonance
OSEM: Ordered subset expectation maximization
PET: Positron emission tomography
PPV: Positive predictive value
RE: Radioembolization
Rratio: Right LPT volume ratio
RtoW: Right LPT to entire liver volume
RV: Relative volume
SIRT: Selective internal radiation therapy
SPECT: Single-photon emission-computed tomography
SUV: Standardized uptake value
TARE: Transarterial RE
TPR: True positive ratio
Vdiff: Volume difference
VOI: Volume of interest

## References

[1] Hendlisz A, den Eynde MV, Peeters M, Maleux G, Lambert B, Vannoote J (2010). Phase III trial comparing protracted intravenous fluorouracil infusion alone or with yttrium-90 resin microspheres radioembolization for liver-limited metastatic colorectal cancer refractory to standard chemotherapy. J Clin Oncol.

[2] van Hazel GA, Heinemann V, Sharma NK, Findlay MPN, Ricke J, Peeters M (2016). SIRFLOX: randomized phase III trial comparing first-line mFOLFOX6 (plus or minus bevacizumab) versus mFOLFOX6 (plus or minus bevacizumab) plus selective internal radiation therapy in patients with metastatic colorectal cancer. J Clin Oncol.

[3] Rognoni C, Ciani O, Sommariva S, Facciorusso A, Tarricone R, Bhoori S (2015). Trans-arterial radioembolization in intermediate-advanced hepatocellular carcinoma: systematic review and meta-analyses. Oncotarget.

[4] Lobo L, Yakoub D, Picado O, Ripat C, Pendola F, Sharma R (2016). Unresectable hepatocellular carcinoma: radioembolization versus chemoembolization: a systematic review and meta-analysis. Cardiovasc Interv Radiol.

[5] Jia Z, Jiang G, Zhu C, Wang K, Li S, Qin X (2017). A systematic review of yttrium-90 radioembolization for unresectable liver metastases of melanoma. Eur J Radiol.

[6] Van Cutsem E, Cervantes A, Adam R, Sobrero A, Van Krieken JH, Aderka D (2016). ESMO consensus guidelines for the management of patients with metastatic colorectal cancer. Ann Oncol.

[7] Vogel A, Cervantes A, Chau I, Daniele B, Llovet J, Meyer T (2018). Hepatocellular carcinoma: ESMO Clinical Practice Guidelines for diagnosis, treatment and follow-up *†*. Ann Oncol.

[8] Bastiaannet R, Kappadath SC, Kunnen B, Braat AJAT, Lam MGEH, de Jong HWAM (2018). The physics of radioembolization. EJNMMI Phys.

[9] Kolligs FT, Bilbao JI, Jakobs T, Iñarrairaegui M, Nagel JM, Rodriguez M (2015). Pilot randomized trial of selective internal radiation therapy vs. chemoembolization in unresectable hepatocellular carcinoma. Liver Int.

[10] Ricke J, Bulla K, Kolligs F, Peck-Radosavljevic M, Reimer P, Sangro B (2015). Safety and toxicity of radioembolization plus Sorafenib in advanced hepatocellular carcinoma: analysis of the European multicentre trial SORAMIC. Liver Int.

[11] Lewandowski RJ, Andreoli JM, Hickey R, Kallini JR, Gabr A, Baker T (2016). Angiogenic response following radioembolization: results from a randomized pilot study of yttrium-90 with or without Sorafenib. J Vasc Interv Radiol.

[12] Padia SA, Lewandowski RJ, Johnson GE, Sze DY, Ward TJ, Gaba RC (2017). Radioembolization of hepatic malignancies: background, quality improvement guidelines, and future directions. J Vasc Interv Radiol.

[13] Dezarn WA, Cessna JT, DeWerd LA, Feng W, Gates VL, Halama J (2011). Recommendations of the American Association of Physicists in Medicine on dosimetry, imaging, and quality assurance procedures for 90 Y microsphere brachytherapy in the treatment of hepatic malignancies. Med Phys.

[14] Ibrahim SM, Lewandowski RJ, Ryu RK, Sato KT, Gates VL, Mulcahy MF (2008). Radiographic response to yttrium-90 radioembolization in anterior versus posterior liver segments. Cardiovasc Interv Radiol.

[15] Mahnken AH, Spreafico C, Maleux G, Helmberger T, Jakobs TF (2013). Standards of practice in transarterial radioembolization. Cardiovasc Interv Radiol.

[16] De Gersem R, Maleux G, Vanbilloen H, Baete K, Verslype C, Haustermans K (2013). Influence of time delay on the estimated lung shunt fraction on 99mTc-labeled MAA scintigraphy for 90Y microsphere treatment planning. Clin Nucl Med.

[17] Minarik D, Sjögreen Gleisner K, Ljungberg M (2008). Evaluation of quantitative 90Y SPECT based on experimental phantom studies. Phys Med Biol.

[18] Selwyn RG, Nickles RJ, Thomadsen BR, DeWerd LA, Micka JA (2007). A new internal pair production branching ratio of 90Y: the development of a non-destructive assay for 90Y and 90Sr. Appl Radiat Isot.

[19] Maughan NM, Eldib M, Faul D, Conti M, Elschot M, Knešaurek K (2018). Multi institutional quantitative phantom study of yttrium-90 PET in PET/MRI: the MR-QUEST study. EJNMMI Phys.

[20] Yue J, Mauxion T, Reyes DK, Lodge MA, Hobbs RF, Rong X (2016). Comparison of quantitative Y-90 SPECT and non-time-of-flight PET imaging in post-therapy radioembolization of liver cancer. Med Phys.

[21] Wright CL, Binzel K, Zhang J, Wuthrick EJ, Knopp MV (2017). Clinical feasibility of 90Y digital PET/CT for imaging microsphere biodistribution following radioembolization. Eur J Nucl Med Mol Imaging.

[22] Gray BN, Burton MA, Kelleher D, Klemp P, Matz L (1990). Tolerance of the liver to the effects of yttrium-90 radiation. Int J Radiat Oncol Biol Phys.

[23] Welsh JS, Kennedy AS, Thomadsen B (2006). Selective internal radiation therapy (SIRT) for liver metastases secondary to colorectal adenocarcinoma. Int J Radiat Oncol Biol Phys.

[24] Garin E, Lenoir L, Rolland Y, Edeline J, Mesbah H, Laffont S (2012). Dosimetry based on 99mTc-macroaggregated albumin SPECT/CT accurately predicts tumor response and survival in hepatocellular carcinoma patients treated with 90Y-loaded glass microspheres: preliminary results. J Nucl Med.

[25] Kao YH (2014). General theory of predictive dosimetry for yttrium-90 radioembolization to sites other than the liver. Cardiovasc Interv Radiol.

[26] Högberg J, Rizell M, Hultborn R, Svensson J, Henrikson O, Mölne J (2015). Increased absorbed liver dose in Selective Internal Radiation Therapy (SIRT) correlates with increased sphere-cluster frequency and absorbed dose inhomogeneity. EJNMMI Phys.

[27] Flamen P, Hendlisz A, Vanderlinden B (2012). Selective internal radiation therapy simulation using 99mTc-labelled macroaggregates of albumin and SPECT-CT. Eur J Cancer Suppl.

[28] Garin E, Rolland Y, Laffont S, Edeline J (2016). Clinical impact of 99mTc-MAA SPECT/CT-based dosimetry in the radioembolization of liver malignancies with 90Y-loaded microspheres. Eur J Nucl Med Mol Imaging.

[29] Pacilio M, Ferrari M, Chiesa C, Lorenzon L, Mira M, Botta F (2016). Impact of SPECT corrections on 3D-dosimetry for liver transarterial radioembolization using the patient relative calibration methodology. Med Phys.

[30] Mikell JK, Mahvash A, Siman W, Mourtada F, Kappadath SC (2015). Comparing voxel-based absorbed dosimetry methods in tumors, liver, lung, and at the liver-lung interface for 90Y microsphere selective internal radiation therapy. EJNMMI Phys.

[31] Pasciak AS, Bourgeois AC, McKinney JM, Chang TT, Osborne DR, Acuff SN (2014). Radioembolization and the dynamic role of 90Y PET/CT. Front Oncol.

[32] Gnesin S, Canetti L, Adib S, Cherbuin N, Silva Monteiro M, Bize P (2016). Partition model-based 99mTc-MAA SPECT/CT predictive dosimetry compared with 90Y TOF PET/CT posttreatment dosimetry in radioembolization of hepatocellular carcinoma: a quantitative agreement comparison. J Nucl Med.

[33] Ljungberg M, Sjögreen Gleisner K (2011). The accuracy of absorbed dose estimates in tumours determined by Quantitative SPECT: a Monte Carlo study. Acta Oncol.

[34] Giammarile F, Muylle K, Delgado Bolton R, Kunikowska J, Haberkorn U, Oyen W (2017). Dosimetry in clinical radionuclide therapy: the devil is in the detail. Eur J Nucl Med Mol Imaging.

[35] Flux GD, Sjogreen Gleisner K, Chiesa C, Lassmann M, Chouin N, Gear J (2018). From fixed activities to personalized treatments in radionuclide therapy: lost in translation?. Eur J Nucl Med Mol Imaging.

[36] Sjögreen Gleisner K, Spezi E, Solny P, Gabina PM, Cicone F, Stokke C, et al. Variations in the practice of molecular radiotherapy and implementation of dosimetry: results from a European survey. EJNMMI Phys. 2017; 4(1):28. 10.1186/s40658-017-0193-4. 10.1186/s40658-017-0193-4 PMC5712507 29199391

[37] Smits MLJ, Elschot M, Sze DY, Kao YH, Nijsen JFW, Iagaru AH (2015). Radioembolization dosimetry: the road ahead. Cardiovasc Intervent Radiol.

[38] Luu HM, Klink C, Niessen W, Moelker A, van Walsum T (2016). Non-rigid registration of liver CT images for CT-guided ablation of liver tumors. PloS ONE.

[39] Carvalho LE, Sobieranski AC, von Wangenheim A. 3D segmentation algorithms for computerized tomographic imaging: a systematic literature review. J Digit Imaging. 2018; 31:1–52. 10.1007/s10278-018-0101-z. 10.1007/s10278-018-0101-z PMC6261188 29915942

[40] Moghbel M, Mashohor S, Mahmud R, Saripan MIB (2018). Review of liver segmentation and computer assisted detection/diagnosis methods in computed tomography. Artif Intell Rev.

[41] Lim SJ, Jeong YY, Ho YS (2006). Automatic liver segmentation for volume measurement in CT images. J Vis Commun Image Represent.

[42] Campadelli P, Casiraghi E, Esposito A (2009). Liver segmentation from computed tomography scans: a survey and a new algorithm. Artif Intell Med.

[43] Liao M, qian Zhao Y, Wang W, zhan Zeng Y, Yang Q, Shih FY (2016). Efficient liver segmentation in CT images based on graph cuts and bottleneck detection. Physica Med.

[44] Wu W, Zhou Z, Wu S, Zhang Y (2016). Automatic liver segmentation on volumetric CT images using supervoxel-based graph cuts. Comput Math Meth Med.

[45] Moghbel M, Mashohor S, Mahmud R, Iqbal Bin Saripan M (2016). Automatic liver segmentation on computed tomography using random walkers for treatment planning. EXCLI J.

[46] Massoptier L, Casciaro S. Fully Automatic Liver Segmentation through Graph-Cut Technique. In: 2007 29th Annual International Conference of the IEEE Engineering in Medicine and Biology Society. IEEE: 2007. p. 5243–5246. https://ieeexplore.ieee.org/abstract/document/4353524. 10.1109/IEMBS.2007.4353524. 10.1109/IEMBS.2007.4353524 18003190

[47] Bekes G, Fidrich M, Ruskó L (2009). Automatic segmentation of the liver from multi- and single-phase contrast-enhanced CT images. Med Image Anal.

[48] Wang J, Cheng Y, Guo C, Wang Y, Tamura S (2016). Shape–intensity prior level set combining probabilistic atlas and probability map constrains for automatic liver segmentation from abdominal CT images. Int J CARS.

[49] Chartrand G, Cresson T, Chav R, Gotra A, Tang A, DeGuise J. SEMI-automated liver CT segmentation using Laplacian meshes. In: 2014 IEEE 11th International Symposium on Biomedical Imaging (ISBI). IEEE: 2014. p. 641–644. https://ieeexplore.ieee.org/abstract/document/6867952. 10.1109/ISBI.2014.6867952.

[50] Shi C, Cheng Y, Liu F, Wang Y, Bai J, Tamura S (2016). A hierarchical local region-based sparse shape composition for liver segmentation in CT scans. Pattern Recognit.

[51] Slagmolen P, Elen A, Seghers D, Loeckx D, Maes F, Haustermans K. Atlas based liver segmentation using nonrigid registration with a B-spline transformation model. In: Proceedings of MICCAI workshop on 3D segmentation in the clinic: a grand challenge.Citeseer: 2007. p. 197–206. http://citeseerx.ist.psu.edu/viewdoc/download?doi=10.1.1.164.6975&rep=rep1&type=pdf. http://mbi.dkfz-heidelberg.de/grand-challenge2007/sites/proceed.htm.

[52] Okada T, Shimada R, Hori M, Nakamoto M, Chen YW, Nakamura H (2008). Automated segmentation of the liver from 3D CT images using probabilistic atlas and multilevel statistical shape model. Acad Radiol.

[53] Heimann T, Wolf I, Meinzer HP (2006). Active shape models for a fully automated 3D segmentation of the liver–an evaluation on clinical data. Med Image Comput Comput-Assist Interv: MICCAI Int Conf Med Image Comput Comput-Assist Interv.

[54] Erdt M, Steger S, Kirschner M, Wesarg S. Fast automatic liver segmentation combining learned shape priors with observed shape deviation. In: 2010 IEEE 23rd International Symposium on Computer-Based Medical Systems (CBMS). IEEE: 2010. p. 249–254. https://ieeexplore.ieee.org/abstract/document/6042650. 10.1109/CBMS.2010.6042650.

[55] Li C, Li A, Wang X, Feng D, Eberl S, Fulham M. A new statistical and Dirichlet integral framework applied to liver segmentation from volumetric CT images. In: 2014 13th International Conference on Control Automation Robotics Vision (ICARCV). IEEE: 2014. p. 642–7. https://ieeexplore.ieee.org/document/7064379. 10.1109/ICARCV.2014.7064379.

[56] Christ PF, Elshaer MEA, Ettlinger F, Tatavarty S, Bickel M, Bilic P (2016). Automatic liver and lesion segmentation in CT using cascaded fully convolutional neural networks and 3D conditional random fields. Evol Int J Org Evol.

[57] Lu F, Wu F, Hu P, Peng Z, Kong D (2017). Automatic 3D liver location and segmentation via convolutional neural network and graph cut. Int J CARS.

[58] Dou Q, Chen H, Jin Y, Yu L, Qin J, Heng PA (2016). 3D deeply supervised network for automatic liver segmentation from CT volumes. Lect Notes Comput Sci (Including Subseries Lecture Notes in Artificial Intelligence and Lecture Notes in Bioinformatics).

[59] Ben-Cohen A, Diamant I, Klang E, Amitai M, Greenspan H. Fully convolutional network for liver segmentation and lesions detection. In: Deep Learning and Data Labeling for Medical Applications, vol. 10008 LNCS. Springer International Publishing: 2016. p. 77–85. https://link.springer.com/chapter/10.1007/978-3-319-46976-8_9. 10.1007/978-3-319-46976-8_9.

[60] Monsky WL, Garza AS, Kim I, Loh S, Lin TC, Li CS (2011). Treatment planning and volumetric response assessment for yttrium-90 radioembolization: semiautomated determination of liver volume and volume of tumor necrosis in patients with hepatic malignancy. Cardiovasc Interv Radiol.

[61] Goryawala M, Guillen MR, Cabrerizo M, Barreto A, Gulec S, Barot TC (2012). A 3-D liver segmentation method with parallel computing for selective internal radiation therapy. IEEE Trans Inf Technol Biomed.

[62] Goryawala M, Gulec S, Bhatt R, McGoron AJ, Adjouadi M (2014). A low-interaction automatic 3D liver segmentation method using computed tomography for selective internal radiation therapy. BioMed Res Int.

[63] Wang X, Li C, Fulham M, Eberl S, Feng D. PET-enhanced liver segmentation for CT images from combined PET-CT scanners. In: 2009 IEEE Nuclear Science Symposium Conference Record (NSS/MIC): 2009. p. 2756–9. 10.1109/NSSMIC.2009.5401962.

[64] Li C, Wang X, Chen J, Yin Y, Feng D. PET-guided liver segmentation for low-contrast CT via regularized Chan-Vese model. In: Proceedings of 2012 IEEE-EMBS International Conference on Biomedical and Health Informatics: Global Grand Challenge of Health Informatics, BHI 2012. IEEE: 2012. p. 816–819. https://ieeexplore.ieee.org/document/6211710. 10.1109/BHI.2012.6211710.

[65] Li C, Wang X, Xia Y, Eberl S, Yin Y, Feng DD (2012). Automated PET-guided liver segmentation from low-contrast CT volumes using probabilistic atlas. Comput Methods Prog Biomed.

[66] Mendes D, Ferreira N, Silva J, Caramelo F. 3D liver segmentation in computed tomography and positron emission tomography exams through active surfaces. In: 2015 IEEE 4th Portuguese Meeting on Bioengineering (ENBENG). IEEE: 2015. p. 1–6. https://ieeexplore.ieee.org/abstract/document/7088895. 10.1109/ENBENG.2015.7088895.

[67] Spahr N, Schilling P, Thoduka S, Abolmaali N, Schenk A (2017). Predictive SIRT dosimetry based on a territorial model. EJNMMI Phys.

[68] Floridi C, Radaelli A, Abi-Jaoudeh N, Grass M, Grass M, Lin M (2014). C-arm cone-beam computed tomography in interventional oncology: technical aspects and clinical applications. La Radiol Med.

[69] Derbel H, Kobeiter H, Pizaine G, Ridouani F, Luciani A, Radaelli A (2018). Accuracy of a cone-beam CT virtual parenchymal perfusion algorithm for liver cancer targeting during intra-arterial therapy. J Vasc Interv Radiol.

[70] Giammarile F, Bodei L, Chiesa C, Flux G, Forrer F, Kraeber-Bodere F (2011). EANM procedure guideline for the treatment of liver cancer and liver metastases with intra-arterial radioactive compounds. Eur J Nucl Med Mol Imaging.

[71] Sietex Medical Limited. SIR-Spheres microspheres (training program/Physicians and Instituions): Sirtex; 2015, pp. 1–111. http://www.westernesse.com/portfoliolks/projects/sirtex/site/pdfs/sir-spheres_user_manual.pdf.

[72] Zeintl J, Vija AH, Yahil A, Hornegger J, Kuwert T (2010). Quantitative accuracy of clinical 99mTc SPECT/CT using ordered-subset expectation maximization with 3-dimensional resolution recovery, attenuation, and scatter correction. J Nucl Med.

[73] Gil-Alzugaray B, Chopitea A, Iñarrairaegui M, Bilbao JI, Rodriguez-Fraile M, Rodriguez J (2013). Prognostic factors and prevention of radioembolization-induced liver disease. Hepatology.

[74] Bastiaannet R, Viergever MA, de Jong HWAM (2017). Impact of respiratory motion and acquisition settings on SPECT liver dosimetry for radioembolization. Med Phys.

[75] De Moor K, Nuyts J, Plessers L, Stroobants S, Maes F, Dupont P. Non-rigid registration with position dependent rigidity for whole body PET follow-up studies. In: 2006 IEEE Nuclear Science Symposium Conference Record, vol. 6: 2006. p. 3502–3506. https://ieeexplore.ieee.org/document/4179797. 10.1109/NSSMIC.2006.353755.

[76] Christensen GE, Joshi SC, Miller MI (1997). Volumetric transformation of brain anatomy. IEEE Trans Med Imaging.

[77] Oliveira FPM, Tavares JMRS (2014). Medical image registration: a review. Comput Methods Biomech Biomed Eng.

[78] Firouzian A, Kelly MD, Declerck JM (2014). Insight on automated lesion delineation methods for PET data. EJNMMI Res.

[79] Cheebsumon P, Yaqub M, van Velden FHP, Hoekstra OS, Lammertsma AA, Boellaard R (2011). Impact of [18F]FDG PET imaging parameters on automatic tumour delineation: need for improved tumour delineation methodology. Eur J Nucl Med Mol Imaging.

[80] Temmerman F, Ho TA, Vanslembrouck R, Coudyzer W, Billen J, Dobbels F (2015). Lanreotide Reduces liver volume, but might not improve muscle wasting or weight loss, in patients with symptomatic polycystic liver disease. Clin Gastroenterol Hepatol.

[81] Taha AA, Hanbury A (2015). Metrics for evaluating 3D medical image segmentation: analysis, selection, and tool. BMC Med Imaging.

[82] Heimann T, Van Ginneken B, Styner MA, Arzhaeva Y, Aurich V, Bauer C (2009). Comparison and evaluation of methods for liver segmentation from CT datasets. IEEE Trans Med Imaging.

[83] Ho S, Lau WY, Leung TWT, Chan M, Ngar YK, Johnson PJ (1996). Partition model for estimating radiation doses from yttrium-90 microspheres in treating hepatic tumours. Eur J Nucl Med.

[84] Gibbs P, Gebski V, Van Buskirk M, Thurston K, Cade DN, Van Hazel GA (2014). Selective Internal Radiation Therapy (SIRT) with yttrium-90 resin microspheres plus standard systemic chemotherapy regimen of FOLFOX versus FOLFOX alone as first-line treatment of non-resectable liver metastases from colorectal cancer: the SIRFLOX study. BMC Cancer.

